# Object detection for automatic cancer cell counting in zebrafish xenografts

**DOI:** 10.1371/journal.pone.0260609

**Published:** 2021-11-29

**Authors:** Carina Albuquerque, Leonardo Vanneschi, Roberto Henriques, Mauro Castelli, Vanda Póvoa, Rita Fior, Nickolas Papanikolaou

**Affiliations:** 1 Nova Information Management School (NOVA IMS), Universidade Nova de Lisboa, Lisboa, Portugal; 2 Computational Clinical Imaging Group, Center for the Unknown, Champalimaud Foundation, Lisboa, Portugal; University Tunku Abdul Rahman, MALAYSIA

## Abstract

Cell counting is a frequent task in medical research studies. However, it is often performed manually; thus, it is time-consuming and prone to human error. Even so, cell counting automation can be challenging to achieve, especially when dealing with crowded scenes and overlapping cells, assuming different shapes and sizes. In this paper, we introduce a deep learning-based cell detection and quantification methodology to automate the cell counting process in the zebrafish xenograft cancer model, an innovative technique for studying tumor biology and for personalizing medicine. First, we implemented a fine-tuned architecture based on the Faster R-CNN using the Inception ResNet V2 feature extractor. Second, we performed several adjustments to optimize the process, paying attention to constraints such as the presence of overlapped cells, the high number of objects to detect, the heterogeneity of the cells’ size and shape, and the small size of the data set. This method resulted in a median error of approximately 1% of the total number of cell units. These results demonstrate the potential of our novel approach for quantifying cells in poorly labeled images. Compared to traditional Faster R-CNN, our method improved the average precision from 71% to 85% on the studied data set.

## Introduction

Cancer caused 10 million deaths in 2020 and is ranked as the sixth leading cause of death worldwide. Moreover, approximately one in five people worldwide develop cancer during their lifetime, and by 2040, the burden of cancer is projected to increase by 47% [1]. Despite the recent advances in this field, cancer treatment usually follows a “one-size-fits-all” approach, which leads to a good response for some patients but not for all. Many patients undergo through trial and error treatment and are subjected to needless toxicity in pursuit of the best treatment. Targeted cancer treatment is a response to this inefficiency; however, methods for predicting how a particular cancer will behave to a specific treatment are still lacking [2]. In evaluating cancer treatments, the zebrafish larvae xenograft is a promising assay for identifying which therapies can lead to better results for precise and targeted medicine [2–5]. With this method, patients do not need to undergo various rounds of treatment based on guidelines to find the best treatment option. Instead, zebrafish larvae xenografts are used as cancer behavior sensors, representing a potential approach to assessing tumor response to treatment in just four days [2, 6, 7]. During this process, the number of cancer cells must be quantified to measure resistance or sensitivity to anti-cancer treatments. However, the cell-counting process is a bottleneck. Due to the cells’ morphology, simple pre-processing techniques do not allow for an accurate counting of the cells; instead, the process requires manual counting, which is a laborious, time-consuming, prone to human error [8, 9]. Furthermore, manual cell counting is a subjective process due to its considerable inter-observer and intra-observer variability [10]. Consequently, automated cell quantification is a long-awaited tool among researchers, and various techniques have been created in recent years to address this problem.

With the advent of deep learning (DL) and convolutional neural networks (CNNs), several authors proposed various techniques that surpassed most traditional approaches. Automatic cell counting can be divided into two major categories: detection-based counting, which demands prior detection or segmentation, and another method based on density estimation or regression without the need for prior labeling [8, 11].

Most cell counting tools developed through the usage of DL are based on recurring object segmentation in frameworks such as Mask R-CNN [12, 13], U-NET [14–16], or feature pyramid networks [17]. However, this type of approach demands pixel-wise labeling of the ground truth, which usually is difficult to obtain.

Another well-explored category is cell counting using regression networks [8, 11, 18, 19], in which the global cell count is taken as an annotation to supervise training, instead of the counting following a classification or detection framework. In this case, there is no need for prior object detection or segmentation as the ground truth. However, the spatial locations of the objects of interest are not identified.

Object detection in a cell quantification context is still poorly explored. The YOLO method has been applied to counting blood cells [20]; however, this system privileges speed over accuracy [21], and it is not sufficiently reliable for use in the medical field. The faster regions of CNN (Faster R-CNN) method has been used on malaria images [22], low-contrast microscopic images [23], and even breast cancer histopathology images [24], demonstrating the system’s potential as an accurate cell counting method. However, Faster R-CNN has mainly been applied to analyze images with negligible cell overlapping and numbers of small objects.

To our knowledge, accurate quantification of single cells in zebrafish xenografts and zebrafish avatars is still highly demanding. Several available cell-counting plugins produce satisfactory performance in two dimensions, but they fail to obtain acceptable results in three dimensions. This is the case of zebrafish xenografts, as well as organoids or spheroids for which these existing systems are ineffective. Furthermore, automatic segmentation tools cannot differentiate objects based on more than the shape, size, or other basic cell features. At the same time, techniques such as intensity thresholds and watershed methods fail when the cell overlap is significantly high, and the nuclei of different cells are located close to one another. This limitation creates a demand for more advanced techniques, and DL systems appear to be reasonable candidates. In 2015 [25], a supervised max-pooling CNN was trained to detect cell pixels in regions that were preselected by a support vector machine (SVM) for automatic cell detection in zebrafish images. Although this CNN performed significantly better than the traditional approaches, the results still showed significant room for improvement, which can be addressed using more complex networks.

In this work we used a detection approach by applying the Faster R-CNN algorithm with the Inception ResNet V2 feature extractor (see S1 Fig) for cell quantification, focusing on accuracy. We conducted experiments using a data set of zebrafish xenografts provided by the Champalimaud Foundation of Lisbon, Portugal. Due to the cell morphology in the provided images, simple pre-processing techniques do not allow for accurate cell counts, and poor labeling prevents the development of a segmentation process. Our experiments focused on optimizing the cell-quantification process. The results indicate that the employed method can achieve promising detection performance, despite the dense cell overlap, the heterogeneity of the cell morphology, and the reduced sample size.

### Main contributions

We present a fully automatic approach for detecting and counting cancer cells in zebrafish xenograft tumor images(see S2 Fig), with the following main contributions:

1. We demonstrate the superior capability of Faster R-CNN compared to single shot detector (SSD), You Only Look Once (YOLO) and region-based fully convolutional networks (RFCNs) as a detection and classification system for complex imaging, when accuracy is a major concern.
2. We comprehensively analyze the parameters of Faster R-CNN that can influence the ability to detect small objects, a common problem in the medical imaging research field.
3. We evaluate the effect of changing the number of proposals when dealing with overcrowded situations and cell overlapping.
4. We demonstrate the potential of data augmentation to increase a network’s performance in given small data sets, which are typical of many medical applications.
5. We analyze the effect of defining accurate anchor rates and scales based on a detailed exploration of the cells, to optimize the process of detecting objects with different shapes.
6. We demonstrate our system’s ability to detect cancer cells in images featuring several problems, by refining the system and adjusting it to address the problems at hand. In this way, we prove the suitability of object-detection frameworks as automatic cell-counting tools for problems in which segmentation is impossible due to inadequate labeling.

Furthermore, we present an improved version of Faster R-CNN, with precise fine-tuning that can handle common issues such as overlapping, small object size, and small data sets in medical imaging. This contribution should encourage further research, beyond the specific data used here.

## Previous and related work

### Object-detection algorithms

In recent years, various strategies have been used to address object-detection problems. However, comparing the performance of systems in the literature is challenging because they feature different base features extractors, image resolutions, and software and hardware platforms. Nevertheless, these systems can be distinguished by considering the trade-off between speed and accuracy [21].

In this work, we focus on four recent meta-architectures that provide differing trade-offs between speed and accuracy: SSD, YOLO, Faster R-CNN, and RFCNs.

SSD [26] is an architecture that uses a single feed-forward convolutional network to predict classes and anchor offsets without the need for a second classification stage, as Faster R-CNN and RFCN require. This characteristic tends to increase the system’s speed while decreasing the model’s accuracy (which is preferable, for instance, for video object detection). The accuracy can always be increased in each of the meta-architectures exposed by using a more robust feature extractor.

YOLO [27] was originally published in 2016 by Redmon et. al. It was announced as an object detection framework that combines in a single network the problem of object localization and classification. YOLO treats detection as a regression problem, in which the image is divided into a grid and for each grid there is a boxing box confidence and a class probability. Known for its efficiency in real-time detection, YOLO has had several proposed improvements [28–30] over the years. The most recent improvement, YOLO v5, developed by Ultralytics [31] uses a cross stage partial network (CSPNet) [32] as the model backbone and path aggregation network (PANet) [33] as the neck for feature aggregation.

Faster R-CNN [34] was developed based on the architecture of the Fast R-CNN method [35], which, in turn, was based on the regions of CNNs (R-CNN) method [36]. Faster R-CNN comprises two networks: the region-proposal network (RPN), whose primary purpose is to generate a set of proposed regions where objects could be present, and a network that uses the first network’s output to detect objects in those regions.

Finally, RFCN is an approach that should be faster than Faster R-CNN because the former is fully convolutional, with almost all computation shared on the entire image. RFCN uses position-sensitive score maps to address the problem of translation-invariance in image classification and translation-variance in object detection [37].

In parallel with the definition of the object-detection system used, feature extractors also can be chosen. Depending on the problem’s complexity, various feature extractors should be tested and have their performance compared.

Residual networks (ResNets), were introduced with the ResNet50 architecture [38]. This type of network uses so-called skipping connections, also called residual blocks, in which some activations from one layer are fed into a deeper layer. This allows the number of layers in the network to be increased efficiently [39].

GoogLeNet/Inception [40] introduced the concept of inception modules to reduce the number of parameters, even with 22 layers of depth. The network comprises nine inception modules, with a total of 100 layers. The inception modules created micro-architectures within the network’s macro-architecture, where operations happen in parallel, and filters were applied to the output to reduce dimensionality [41].

The Inception ResNet [42] is based on the Inception architecture and introduces residual blocks to the architecture. Szegedy et al. found that Inception ResNet models can achieve higher accuracy values at lower epochs.

The NASNet system [43] was developed to optimize convolutional architectures. Inspired by the neural architecture search (NAS) framework, Zoph et al. [43] developed a system for “searching” the space of network architectures on a small proxy data set (CIFAR-10). Then, the learned architecture was transferred to a larger data set (IMAGENET), using concepts such as architecture scalability and flexibility. The system outperformed a set of human-designed models.

Most state-of-the-art detectors perform well on challenging data sets such as COCO or PASCAL VOC since these data sets typically contain objects taking medium or large parts of an image, and the number of samples is significant. However, most of them struggle to detect overlapping and/or small objects in data sets of small size. In crowded scenes, objects tend to overlap largely with each other, leading to occlusions, and detection boxes with high overlaps can match the same object. In such a situation, it is often appropriate to apply strategies such as, for instance, Non-Max suppression, where the most appropriate bounding box for the object is selected. Additionally, small objects can be difficult to detect due to their low resolution. Moreover, existing deep neural networks lose the features of objects after several convolutions and pooling operations [44]. The approaches to address those problems are typically meta-architecture dependent, since the strategies and hyperparameters used can significantly change from a type of architecture to another. The following subsections refer to some of the used techniques to address this problem by employing Faster R-CNN, the algorithm used in this study.

### Detection of small objects

In recent years, several authors have proposed various solutions to optimize small-object detection in images. Hu et al. [44] extracted image features from their third, fourth, and fifth convolutions and merged those into a one-dimensional vector. Some methods for detecting small objects were suggested using Faster R-CNN or SSD as the background [45].

Eggert et al. [46] presented an improved scheme for generating anchor suggestions and they proposed a modified Faster R-CNN that used higher-resolution feature maps for small objects. In [47], an improved loss function was proposed based on the intersection over union (IoU) for bounding box regression, and bilinear interpolation was used to improve the pooling operation for regions of interest (RoIs), to solve positioning error. In the detection phase, the authors used multiscale convolution feature fusion so that the feature map would contain more information and to improve the non-maximum suppression (NMS) technique in order to avoid the loss of overlapping objects.

Fu et al. [48] added deconvolution layers to SSD+Residual-101 to introduce additional large-scale context into object recognition and improve accuracy, especially for small objects. Cao et al. [49] proposed a multi-level feature-fusion method for introducing context information into SSD to improve accuracy for small objects. In [50], several high-level feature maps at various scales were extrapolated simultaneously to increase the spatial resolution. Tong et al. [51] delved deeper into handling small objects in computer vision. They suggested five perspectives of possible future research directions: emerging small-object-detection data sets and benchmarks, multi-task joint learning and optimization, information transmission, weakly supervised small -object -detection methods, and frameworks for small-object detection. Moreover, the atrous rate is a mechanism that increases the model’s performance when detecting small objects [45]. This rate is applied to the tensor associated with the features to crop in the first stage in order to obtain box predictions.

### Detection in crowded and overlapping scenes

Faster R-CNN outperforms Fast R-CNN by using an RPN with a CNN model, whose primary purpose is to propose a set of regions where objects could be present. By default, Faster R-CNN assumes 2,000 proposals, which are then reduced to a small number of proposals, based on the number of relevant objects detected in the first stage and reshaped to a fixed size in a process called RoI pooling [34]. Because the original version of Faster R-CNN was designed for images with a relatively small number of relevant objects, the network must be adjusted to the new context, in which the number of cells in the image can be relatively high, similar to what is applied for detecting humans in crowded scenarios [52]. This change demands higher precision in adjusting the NMS threshold, which is responsible for selecting the most appropriate bounding box for the detected object, because a higher value than needed can lead to more false positives, and a lower threshold can miss the detection of possibly relevant objects.

### Small data sets

Data augmentation is the process whereby new instances are created from existing ones, usually on the fly, to avoid waste of storage. Several techniques can be applied, such as rotation, zoom, change in channel colors, and change of saturation.

These approaches introduce noise into the training process and force the model to be more tolerant of possible changes to the images, including position, orientation, and size, making the model more robust and mitigating overfitting. This process can be useful when dealing with small data sets, a common problem when analyzing medical images. In fact, it artificially boosts the size of the training set [53]. Several techniques were applied in this work:

1. **Random horizontal flip**—The image and detections were flipped horizontally. This occurred randomly with a 50% probability.
2. **Random vertical flip**—The image and the ground truth annotations were flipped vertically. This occurred randomly with a 50% probability.
3. **Pixel value scale**—The values of all pixels in the image were scaled randomly by a constant value between fixed minimum and maximum values.
4. **Random Image scale**—Images were enlarged or shrunk randomly, while keeping the same aspect ratio.
5. **RGB to gray**—Entire images were converted to grayscale randomly, with a 10% probability.
6. **Adjust brightness**—The image brightness was changed randomly, up to a maximum prefixed threshold. The image outputs were saturated at values between 0 and 1.
7. **Adjust contrast**—The contrast was scaled randomly by a value between fixed minimum and maximum values.
8. **Adjust saturation**—The saturation was altered randomly by a value between fixed minimum and maximum values.
9. **Distort color**—A random color distortion was performed.
10. **Jitter boxes**—The corners of the boxes in the images were jittered randomly, as determined by a ratio. For instance, if a box was [100, 200] and the ratio was 0.02, the corners could move by [1, 4].
11. **Crop image**—The images and bounding boxes were cropped randomly.
12. **Crop to aspect ratio**—The images were cropped randomly to a given aspect ratio.
13. **Black patches**—Black square patches were randomly added to an image.
14. **Rotation 90**—The image and detections were rotated randomly by 90° counter-clockwise 50% of the time.

### Dealing with objects of different shapes

The original version of Faster R-CNN, by default, generated anchor boxes with three aspect ratios (1:1, 1:2, 2:1) and three scales (128 × 128, 256 × 256, 512 × 512), resulting in nine anchor boxes. The anchors can be adjusted to the problem at hand, considering different objects have different shapes, which will affect the algorithm’s performance in the detection stage, as presented in [54], where the anchors were adjusted to detect cars. In the present work, this parameter can be changed within Faster R-CNN to deal with the morphology of the studied cells.

## Materials and methods

### Data

The data we used in this study included 97 RGB images of zebrafish xenografted tumors, acquired with the initial purpose of evaluating targeted cancer treatment and assessing tumor response to various therapies. For this specific study, the images were labeled manually using the Fiji software [55]. The labeling was performed manually by a domain expert at the Champalimaud Foundation, who applied a single dot over each cell.

Among the images, 89 had dimensions of 512 × 512 pixels, and the remaining eight had dimensions of 1280 × 1280 pixels, all with three channels, as a result of immunofluorescence technique, used to label nuclei, human cells and apoptotic or immune cells. These eight images were scaled to 512 × 512 pixels, using the inter-area technique, which is considered the best for decreasing the dimensions of the images [56]. Because the number of cells varied significantly across the images (ranging between 18 to 661), the images were divided into three sections based on the cells’ complexity and density:

- **Low**—Images with fewer than 50 cells;
- **Medium**—Images with cells numbering in the range [250]; and
- **High**—Images with greater than 250 cells.

**Fig 1:**
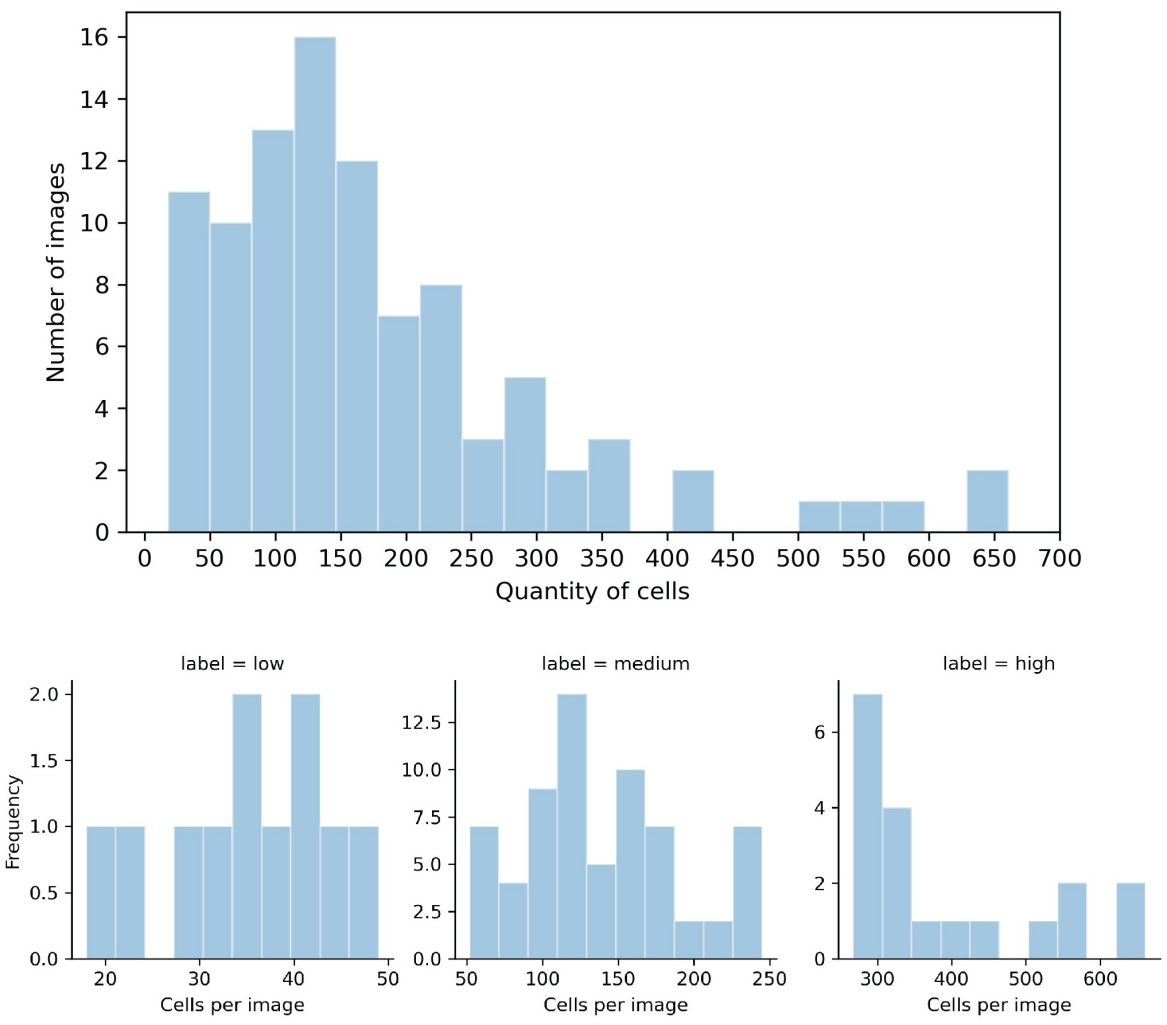
Distributions of the number of cells in the considered images. The top histogram shows the distribution for the whole data set, and the bottom histograms show the distributions for the low, medium, and high levels of complexity. In a range of 18 to 661 cells, the majority of images had at most 250 cells. Only eleven images had fewer than 50 cells, and nineteen images had more than 250.

Fig 1 shows the distribution of the numbers of cells in each image.

The majority of the images had between 50 and 300 cells. Additional descriptive statistics regarding the cell numbers in the raw images are given in Table 1.

**Table 1:** Descriptive summary (mean, range, percentiles, skewness and kurtosis) of the cell quantities in the provided images, for the whole data set and for the various complexity levels.

Images (count)	Mean ± SD	Range [min, max]	25%	50%	75%	Skew	Kurt
All (96)	176.6 ± 132.4	[18,661]	95	137	230	1.7	3.3
Low (11)	35.2 ± 9.1	[18,49]	31	35	42	-0.5	-0.2
Medium (66)	138.9 ± 52.5	[52,245]	104	129	173	0.4	-0.6
High (19)	391.2 ± 131	[267,661]	295	343	471	1.0	-0.3

On average, we had approximately 176 cells per image, but the standard deviation was 132, highlighting the need to define different groups of images according to the number of cells. Defining different groups causes the standard deviation of each group will decrease, as well as its skewness and kurtosis to decrease. Having high values for such elements can lead to underestimation for some predictions.

High-skewness situations are assumed when the value of skewness is less than −1 or greater than 1 [57]. Similar to skewness, high kurtosis values are less than −2 or greater than 2. As shown in Table 2, the whole data set has a skewness of 1.7, and we also had a leptokurtic (kurtosis higher than 3) distribution, which can lead to situations in which the predictive model underestimates some predictions. With that in mind, the data set was partitioned into three sets (low, medium, and high) to reduce skewness and kurtosis for each sample.

**Table 2:** Descriptive summary (mean, range, percentiles, skewness and kurtosis) of the cell dimensions (width, height and ratio).

Dimension	Mean±SD	Range [Min,Max]	25%	50%	75%	Skew	Kurt
Width	15.9±4.5	[4,44]	13	15	19	0.8	1.1
Height	15.2±4.3	[3,45]	12	15	18	0.9	2.8
Ratio	1±0.3	[0.3,3.3]	0.8	0.9	1.1	18.9	0.1

On average, the cells had a width of 15.9 pixels, a height of 15.2 pixels, and a ratio of 1. Nevertheless, their width ranged from 4 to 44 pixels, their height ranged from 3 to 45 pixels, and their ratio ranged from 0.3 to 3.3, demonstrating the cells’ irregular morphology in terms of size and shape. The ratio is calculated by dividing width by height.

Taking into account the small number of samples available, the fine-tuning of the model considered the entire data set, but the regression measures calculated took data set’s partitioning into account.

**Fig 2:**
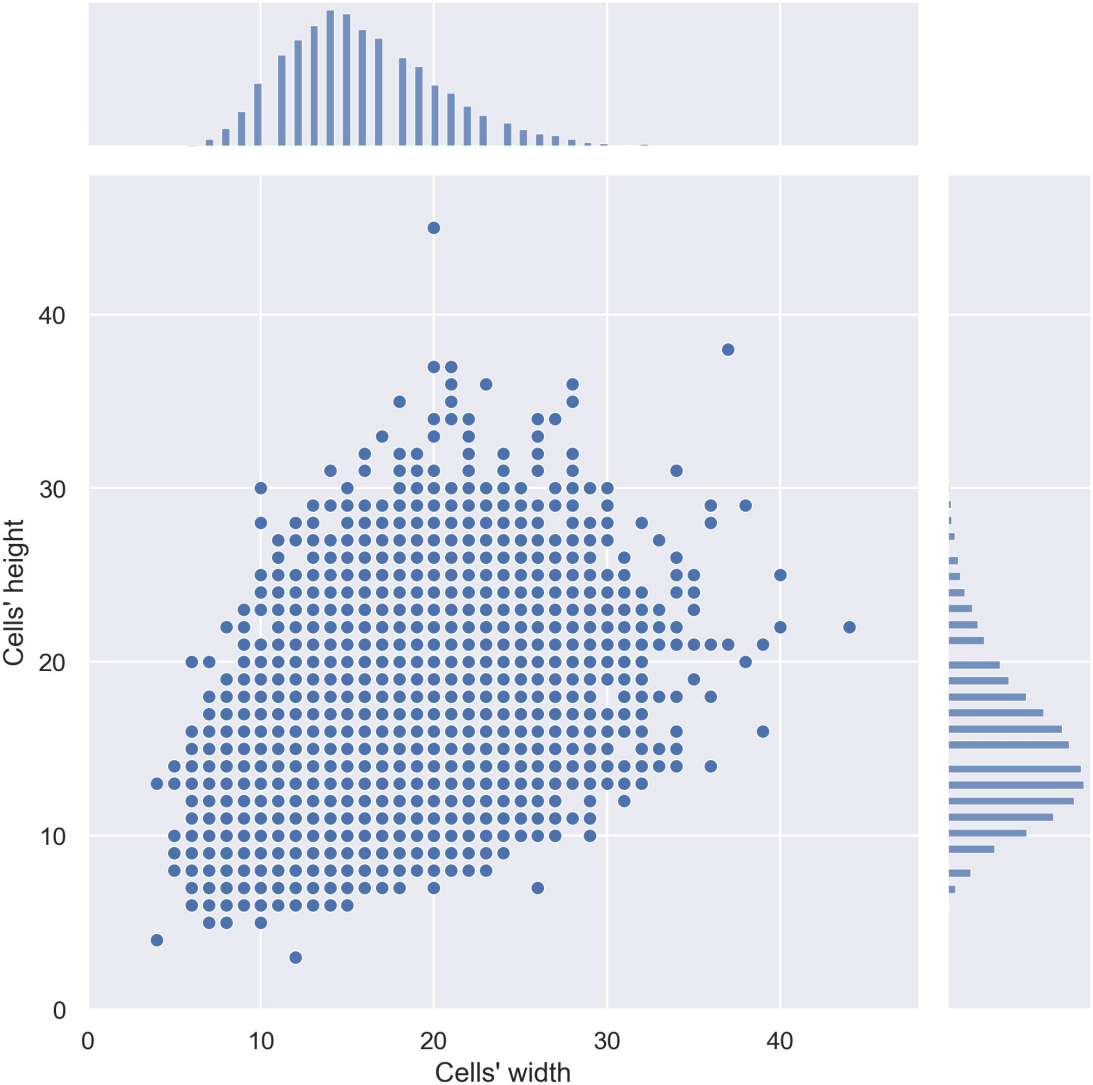
Joint and marginal distribution plot representing the density and distribution of the cells according to their width and height. Although it is normally distributed, we can verify the significant variance of cell morphology available in the data set.

Besides the number of cells present in each image, it was important to establish aspect ratios and scales when generating anchors to evaluate the distributions of the width, height, and aspect ratios of the 17,128 cells present in the images. The statistical measures reported in Table 2, and displayed in Fig 2, refer to images with dimensions 512 x 512 pixels. If the image is resized when fine-tuning the model, the scaling of these values should be considered.

**Fig 3:**
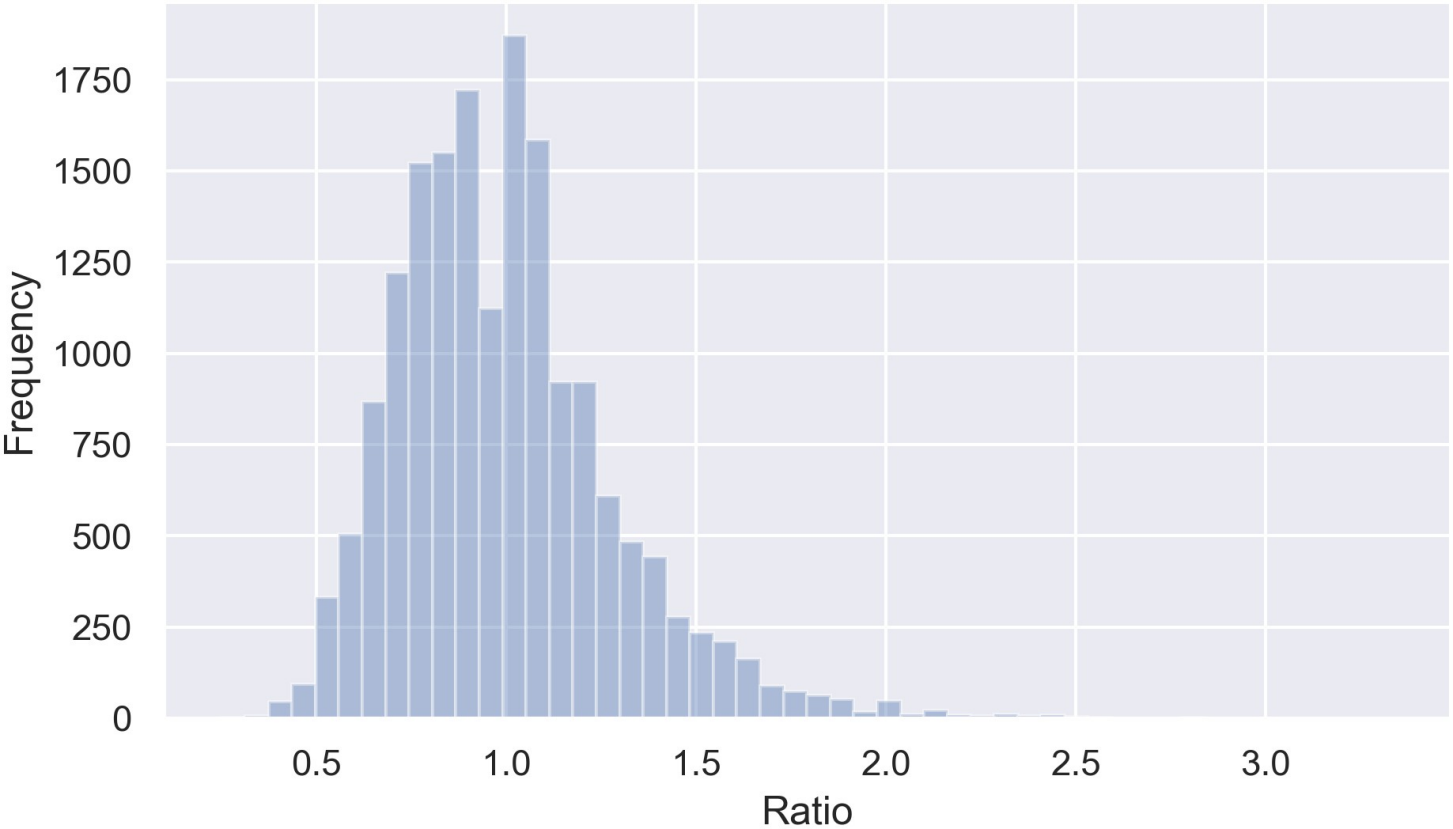
Histogram plot for the frequency of cells according to their ratio (width/height). The cells’ ratio, which can influence the aspect ratio definition in Faster R-CNN, varied between 0.3 and 3.3.

**Fig 4:**
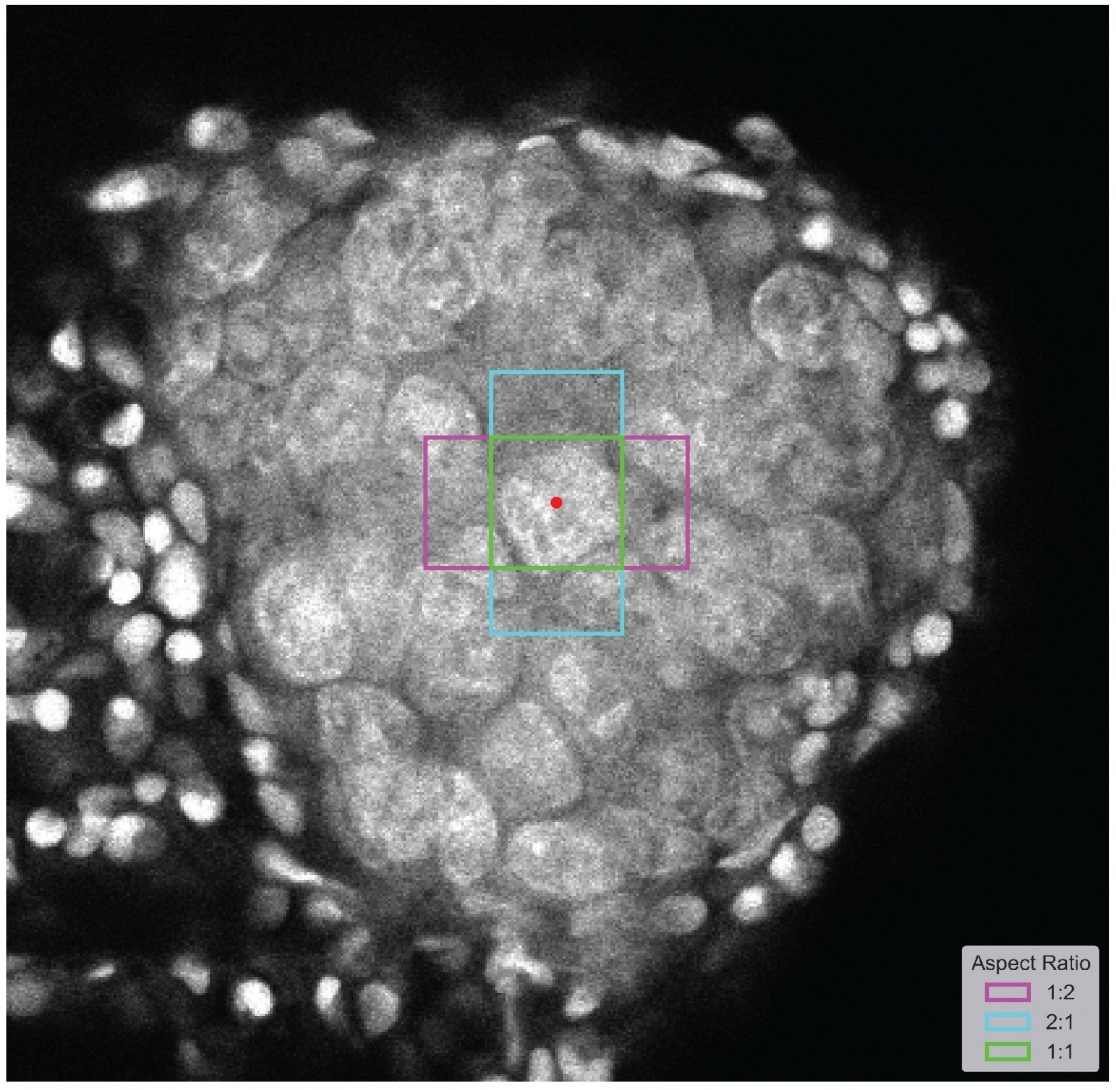
Example of possible aspect ratios, taking into account the ratio distribution. The magenta shape corresponds to a 1:2 aspect ratio, the blue corresponds to a 2:1 aspect ratio, and the green corresponds to a square. The red dot is the anchor.

As shown in Fig 3, the cells’ aspect ratio ranged between 0.5 and 2.0. One possible approach to selecting aspect ratios for the anchor generator was to start with three ratios (e.g., 0.5, 1.0, and 2.0) that cover the majority of the distribution, as in Fig 4.

#### Data set splitting and labeling

To divide the initial data set, we considered not only the number of images but also the number of available cells or objects shown in the various images and the average number of cells in each image for the data sets, as this value could range between 18 and 661 cells.

The initial data set was split into three data sets, taking into account the following proportions: around 80% for training, 15% for validation, and 5% for testing, as shown in Table 3. Considering the data set’s small size, as well as the idea that this work aimed at tuning a network to be able to deal with the several limitations available in the data, we decided to add a more considerable proportion of data to the validation set and a smaller proportion to the test set, the latter of which was used only after we selected the best model.

**Table 3:** Data set partitioning into training, validation and test data sets, considering the quantity of cells and the quantity of images.

Data set	# cells	% cells	# images	% images	Avg. #cells per image
Train	13790	80.5	79	81.3	176.8
Validation	2292	13.4	13	13.5	176.3
Test	1046	6.1	5	5.2	209.2
Total	17128	100	97	100	176.6

The data were partitioned into training, validation, and test data sets. This partition took into consideration the number of cells, the percentage of cells, the number of images, the percentage of images, and the average quantity of cells per image. We used around 80% of the data to train the model, 15% for validation purposes, and 5% for testing.

The labeling of the images in the data set was inadequate for segmentation. Each cell was annotated by a dot, which did not correspond exactly to the center of the cell. To overcome this problem and apply object detection, we performed additional labeling considering those initial annotations using the labelImg software. This software allows bounding boxes to be created around objects to extract the coordinates of those boxes in the image.

#### Loss functions

The loss function in Faster R-CNN is evaluated during the algorithm’s two stages. At each stage, the error is measured using a multi-loss function, which sums up to four losses in total. The first stage of the algorithm, the RPN, measures the model’s performance, taking into account objectness loss and localization loss, which result in the multi-loss function defined in Eq 1:

$$\begin{array}{c} L\left(\{p_{i}\},\{t_{i}\}\right)=\frac{1}{N_{cls}}\sum_{i}^{}L_{cls}\left(p_{i},p_{i}^{*}\right)+\lambda \frac{1}{N_{reg}}\sum_{i}^{}p_{i}^{*}L_{reg}\left(t_{i},t_{i}^{*}\right) \end{array}$$ (1)

Thus, the RPN takes an image (of any size) as its input and outputs a set of rectangular object proposals, each with an objectness score [34]. In Eq 1, *p* stands for the predicted objectness class, p* for the ground truth objectness class, *t* for the ground- truth bounding box, and t* for the predicted bounding box.

Objectness loss classifies a patch as having, or lacking an object. In this phase, the goal is not to identify the class of the identified object but to determine whether a patch contains an object or the background. This objectness score is used to filter the bad predictions in the second stage. With this purpose in mind, Faster R-CNN uses a classifier with two possible classes: one for the presence of an object and one for the background.

Localization loss, in which a regression is applied to the bounding box, considers the parametrization of the four following coordinates, as shown in Eq 2:

$$\begin{array}{c} \begin{array}{c} t_{x}=\frac{\left(x-x_{a}\right)}{w_{a}}\\ t_{w}=\frac{w}{w_{a}}\\ t_{x}^{*}=\frac{\left(x^{*}-x_{a}\right)}{w_{a}}\\ t_{w}^{*}=log\frac{w^{*}}{w_{a}} \end{array}\;\;\;\begin{array}{c} t_{y}=\frac{\left(y-y_{a}\right)}{h_{a}}\\ t_{h}=log\frac{h}{h_{a}}\\ t_{y}^{*}=\frac{\left(y^{*}-y_{a}\right)}{h_{a}}\\ t_{h}^{*}=log\frac{h^{*}}{h_{a}}, \end{array} \end{array}$$ (2)

where *x*, *y*, *w*, and *h* denote the box’s center coordinates and its width and height. The variables *x*, *x*_*a*_, and x* denote the predicted box, anchor box, and ground-truth box respectively (likewise for *y*, *w*, and *h*).

For the second stage of the algorithm, two new losses are computed: the box-predictor localization loss and the box-predictor classification loss. Each RoI is labeled with a ground truth class *u* and a ground-truth bounding-box regression target *v*. The multi-loss function is defined as seen in Eq 3:

$$\begin{array}{c} L\left(p,u,t^{u},v\right)=L_{cls}\left(p,u\right)+\lambda [u\geq 1]L_{loc}\left(t^{u},v\right), \end{array}$$ (3)

where *p* stands for the discrete probability distribution over the categories and *t*^*u*^ is the bounding-box regression achieved from the first stage using the RPN localization loss. The indicator function [*u* ≥ 1] evaluates to 1 when u ≥ 1 and 0 otherwise (the background is labeled as 0). The hyperparameter λ controls the balance between the two losses [35].

*Box-predictor localization loss*. Ren et al. [34] applied a smooth-L1 loss on the position (x,y) of the top-left of the bounding box and the logarithm of the heights and widths, similar to the RPN localization loss applied on the first stage, as shown in Eq 4:

$$\begin{array}{c} L_{Loc}\left(t^{u},v\right)=\sum_{i\epsilon x,y,w,h}^{}{\text{smooth}}_{L_{1}}\left(t_{i}^{u}-v_{i}\right), \end{array}$$ (4)

where

$${\text{smooth}}_{L_{1}}\left(x\right)=\{\begin{array}{cc} 0.5x^{2} & \text{if}|x|<1\\ |x|-0.5 & \text{otherwise} \end{array}$$

where *x* is the difference between the predictions and target.

Choosing a smooth L1 loss comes from the fact that this type of loss is less sensitive to outliers and thus is more robust. When the regression targets are unbounded, training with L2 loss can demand careful tuning of the learning rates to avoid exploding gradients [35].

This loss is aimed at refining the localization of the bounding boxes before classification.

*Box-predictor classification loss*. By default, weighted sigmoid was used to quantify classification loss. However, focal sigmoid classification loss and bootstrapped sigmoid classification loss were also tested.

As a binary classification, all former classification losses were based on the cross-entropy loss for binary classification [58], as presented in Eq 5:

$$\begin{array}{c} CE\left(p,y\right)=\{\begin{array}{cc} -log(p) & \text{if}\;y=1\\ -log(1-p) & \text{otherwise} \end{array} \end{array}$$ (5)

In Eq 5, *y* ∈ {±1} specifies the ground truth class and *p* ∈ [0, 1] is the model’s estimated probability for the class with label *y* = 1. For notational convenience, *p*_*t*_ is defined as follows:

$$\begin{array}{c} p_{t}=\{\begin{array}{cc} p & \text{if}\;y=1\\ 1-p & \text{otherwise} \end{array} \end{array}$$ (6)

and

$$\begin{array}{c} CE\left(p,y\right)=CE\left(p_{t}\right)=-log\left(p_{t}\right) \end{array}$$ (7)

#### Evaluation metrics

Mean average precision (mAP) is the standard metric used to evaluate an object-detection algorithm. This metric is the product of the precision and recall of the detected bounding boxes and ranges from 0 to 1, with higher values indicating better performance. The mAP, which is based on the concept of IoU, is a good measure of the network’s sensitivity [41]. The IoU is the ratio of the overlapping area between the ground truth and predicted area to the total area or union area.

In addition to the mAP metric, used to fine-tune the model, and taking into account the problem in hand, other metrics were considered to evaluate the model’s performance. Although mAP is the standard metric used in object-detection systems, it has limitations, especially when dealing with a large number of objects. This happens because if a specific object is not identified on a region proposal at the first stage of the algorithm, it will not be taken into account for the model’s accuracy. In this way, each model’s performance in the validation data set was tested, and several metrics were applied to measure the regression performance between the predicted value, (i.e., the number of objects predicted) and the ground-truth value. The root mean squared error (RMSE), mean absolute error (MAE), and median absolute error (MedAE) were taken into account. In the end, due to the nature of the problem, our best models should account for not only the mAP, but also the regression metrics. With that purpose, six champion models were selected and trained for additional epochs. The model that provided the best performance was considered the winner.

#### Implementation details

The experiments were implemented using the TensorFlow Object Detection API, an open-source framework built on top of TensorFlow. All tests described in the following sections were carried out using Azure cloud computing resources, namely four Tesla K80 GPUs (2 physical cards) with 32 GiB GPU memory, on a virtual machine with 24 vCPUs, 224 GiB of memory, and a 1.44 TB SSD as temporary storage.

## Results and discussion

### Performance comparison of different object-detection systems

In this experiment, we tested the performance of different feature extractors and meta-architectures using a model pretrained on the COCO data set [59]. Each model was run for 4,000 steps (200 epochs) with images resized to 600 x 600 pixels, the default value in TensorFlow API.

#### Choosing the meta-architecture

During the initial stage, our concern was defining the best meta-architecture for the problem at hand.

**Fig 5:**
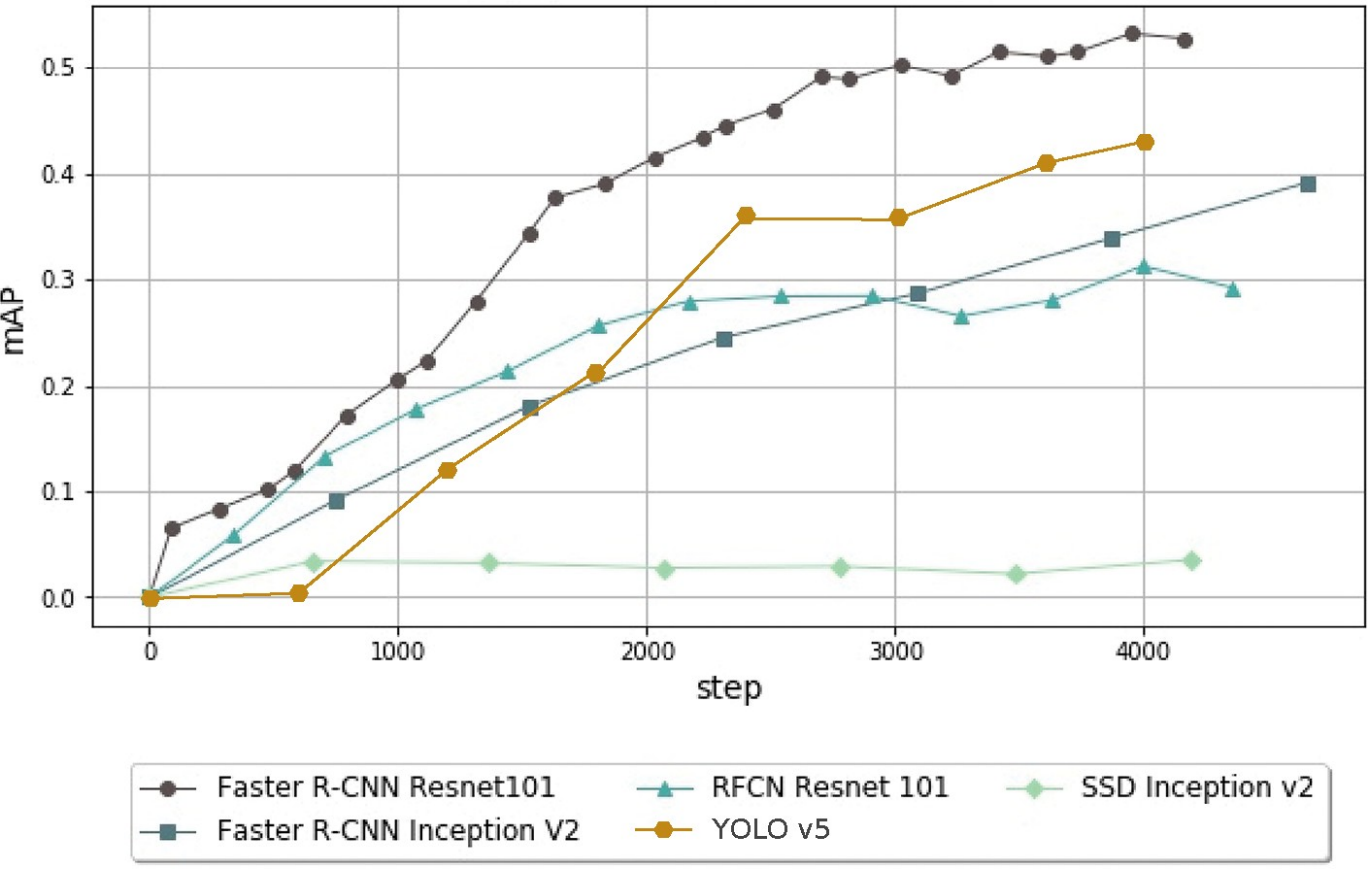
Performance comparison using the mAP of the object-detection models trained using various meta-architectures (Faster R-CNN, SSD, YOLO and RFCN) for 4,000 steps. Faster algorithms such as SSD cannot deal with the complexity of the problem. Faster R-CNN emphasizes accuracy over speed and can achieve over six times better performance than SSD with the same feature extractor. YOLO v5, the last version of YOLO, outperforms SSD and RFCN, but Faster R-CNN still has an advantage of 0.1 mAP at 4,000 steps.

As shown in Fig 5, we created four models using RFCN, SSD, YOLO and Faster R-CNN. The R-CNN model showed a clear advantage over the others. When comparing SSD and Faster R-CNN, and using Inception V2 as a feature extractor, Faster R-CNN achieved a mAP value of 0.34 after 4,000 steps, whereas SSD was unable to exceed 0.04 mAP. When comparing RFCN and Faster R-CNN, this time with ResNet101 as a feature extractor, RFCN achieved a mAP of 0.31 after 4,000 steps, whereas Faster R-CNN achieved a value of 0.53 mAP. When implementing YOLO v5, provided by Ultralytics [31, 60], we achieved a value of 0.43 mAP at 4,000 steps. This experiment shows the suitability of Faster R-CNN for the problem at hand, compared to the other meta-architectures tested.

#### Defining the best feature extractor

To better understand the influence of the feature extractor, we applied five feature extractors to Faster R-CNN: Inception V2, NAS, ResNet50, ResNet101, and Inception ResNet V2.

Table 4 compares the mAP values at the validation data set and the speed of the feature extractors when training the model. At 4,000 steps, when Faster R-CNN achieves convergence, Inception ResNet V2 performed the best, achieving a value of 0.71 mAP. As expected, increasing the feature extractor complexity increases training speed, and when using NAS, this value reaches 96 seconds per epoch, even if the model’s performance is not the highest, with a mAP of 0.44.

**Table 4:** Validation performance and training speed for Inception V2, NAS, ResNet50, ResNet101 and Inception ResNet V2 on Faster R-CNN.

Meta-Architecture	Feature Extractor	mAP at 4,000 Steps	Speed/epoch (sec)
Faster R-CNN	Inception V2	0.34	6.17
Faster R-CNN	NAS	0.44	96.36
Faster R-CNN	ResNet50	0.50	10.34
Faster R-CNN	ResNet101	0.53	47.08
Faster R-CNN	Inception ResNet V2	0.71	45.55

This table presents mAP values at 4,000 steps and time to train an epoch in seconds using Faster R-CNN with different feature extractors (Inception V2, NAS, ResNet50, ResNet101, and Inception ResNet V2). Changing the feature extractor and, consequently, the network complexity, can lead to significant changes in mAP, ranging from 0.34 on Faster R-CNN with Inception V2 to 0.71 on Faster R-CNN with Inception ResNet V2. Increasing the complexity also affects training speed. Clear evidence of this fact can be seen when changing the network from ResNet50, which is 50 layers deep, to ResNet101, which has 101 layers. The mAP increases slightly, but training takes more than four times longer.

These results demonstrate that Faster R-CNN can achieve a performance of around 18 times better when upgrading the feature extractor from Inception V2 to Inception ResNet V2, thus demonstrating the advantage of using the latter when accuracy is the primary goal.

### Performance evaluation for detecting small objects

The purpose of the experiments that followed the testing of the best feature extractor was to increase the system’s capability to detect small cells. Thus, we trained our model (Faster R-CNN with Inception ResNet V2) in various experiments by changing the size of the figure, changing the stride used in the anchor generator, using an atrous rate, and adjusting the IoU threshold. Starting from the default values at TensorFlow API (S1–S3 Tables), grid search was used on those hyperparameters to maximize the model’s performance without overfitting. This tuning process is clarified on the following sections.

#### Changing the image size

Because we are dealing with small objects and considering the known limitations of object-detection algorithms, one possible approach to dealing with this situation is to increase the size of the input images. To test the effect of image resizing on the algorithm’s performance, we conducted experiments using various sizes and resizing methods.

We trained five models for 3,000 steps and changed the input images’ size, where the lowest resolution was 256 × 256 and the highest was 1100 × 1100.

As a default, TensorFlow API assumes that an image should have a minimum of 600 pixels or a maximum of 1024 pixels by mantaining the aspect ratio. By dealing with squared images and knowing that an increase in the number of pixels demands a higher training time and more computational resources, we made several adjustments to understand how the image resizing can affect the algorithm’s performance. At most, we resized the image to a dimension of 1100 × 1100 pixels, the maximum possible size for training the network with a batch of 1 without a RAM leak.

**Fig 6:**
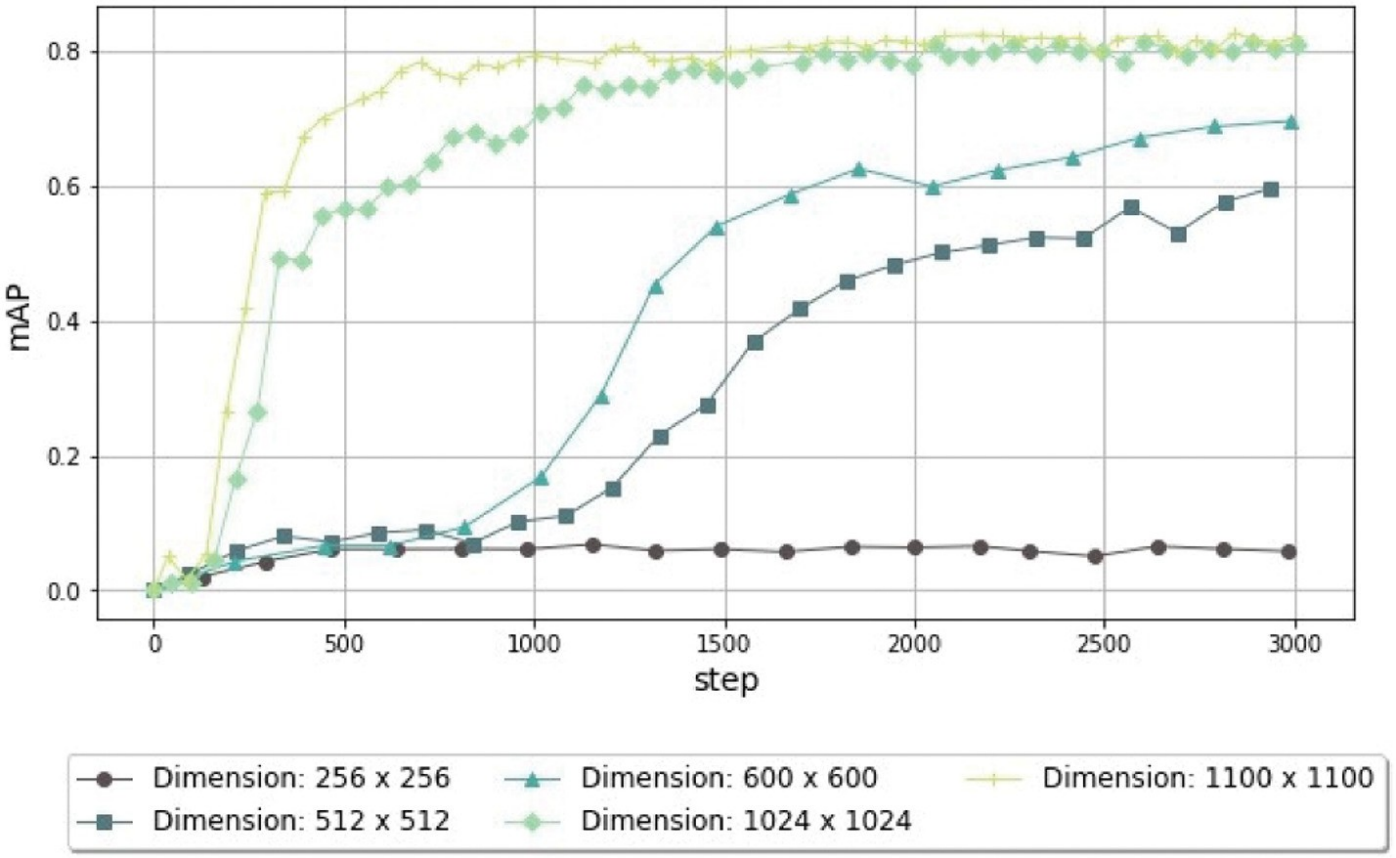
Performance comparison using the mAP of the object-detection models trained using different image sizes (from 256 × 256 pixels to 1100 × 1100 pixels) for 3,000 steps. When dealing with very small cells with a small number of pixels, it is important to increase the resolution of the image to improve the richness of features. In this way, the detector can correctly identify and classify the cells.

As Fig 6 indicates, the images’ dimensions significantly influenced the model’s mAP, probably because of the presence of small objects in the input data. This was corroborated by the results presented in Table 5, which indicate a decrease in the RPN localization loss and RPN objectness loss for the higher-resolution images.

**Table 5:** Comparison of detection results when changing image size from 256 pixels to 1100 pixels.

Size (pixels)	Total Loss Validation	RPN validation loss		mAP	Speed/epoch (sec)
		Loc.	Obj.		
256x256	3.47	2.92	0.63	0.06	28.22
512x512	3.19	1.82	0.29	0.57	38.80
600x600	2.61	1.65	0.16	0.70	45.55
1024x1024	1.86	0.89	0.07	0.81	83.77
1100x1100	2.28	0.82	0.06	0.82	94.13

Total loss for validation, RPN validation loss for localization and objectness, mAP, and speed of training one epoch in seconds for 3,000 steps using different image-input dimensions (from 256 x 256 pixels to 1100 x 1100 pixels). There is a trade-off between accuracy and speed. A higher resolution will allow achieving a better mAP and a slower training time. With an increase of the image resolution from 256 pixels to 1100 pixels, the mAP rose from 0.06 to 0.82.

Furthermore, the dimensions also affected the model’s convergence: when using the original dimensions (512 × 512), the model will began to stabilize only at 3,000 steps. However, when using the maximum dimensions, a convergent state is achieved at around 1,000 steps, as shown in Fig 6. Moreover, it is possible to conclude that low-resolution images do not allow the model’s performance to improve during training, as seen in the images with dimensions of 256 × 256. Finally, as expected, increasing the dimensions of the images also increases the time required to conclude each epoch also increases. This ranged from 28 seconds for the lowest resolution to 94 seconds for the highest one.

**Fig 7:**
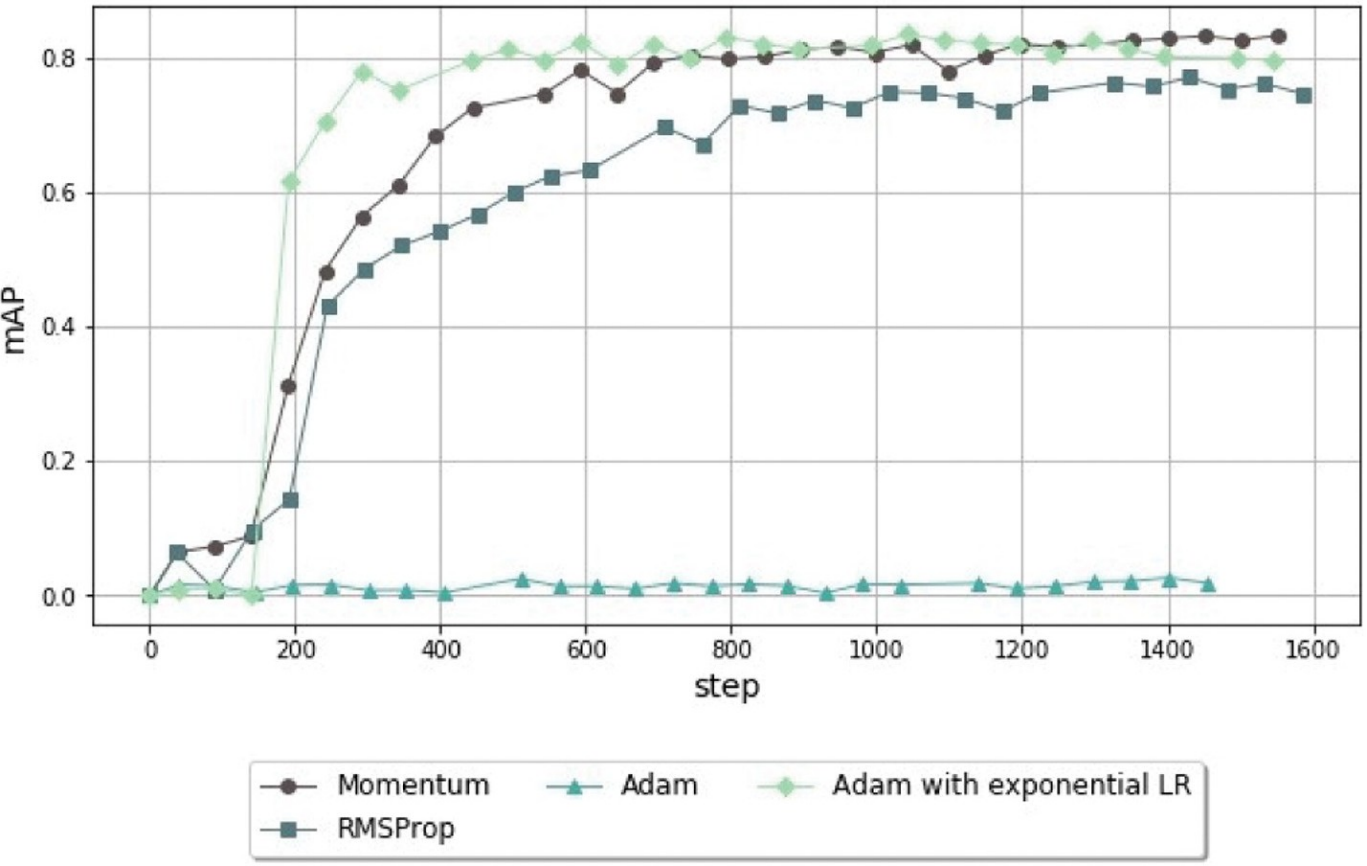
Comparison of mAP performance of the object-detection models trained using different optimizers (Momentum, Adam, Adam with exponential learning rate, and RMSProp) for 1,400 steps. Adam, which is known for achieving a faster convergence than the remaining optimizers, cannot achieve a satisfying performance if the learning rate decay is not well adjusted.

Before running further experiments, we adjusted the optimizer and the learning rate (LR). Whereas the Momentum optimizer was applied in the original Faster R-CNN, we tested three optimization algorithms to check their impact on the model (Fig 7).

**Fig 8:**
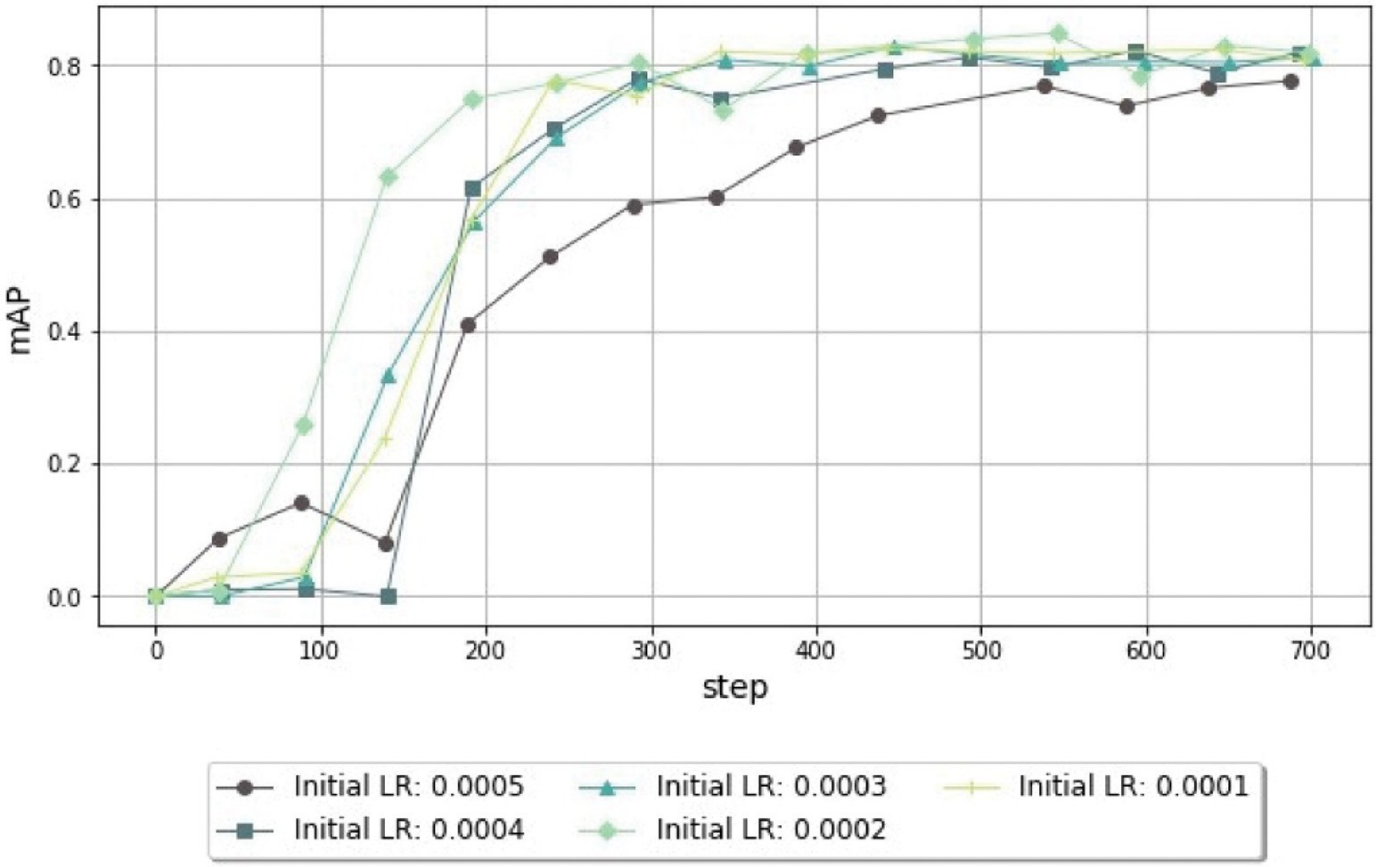
Comparison of mAP values from the object-detection models trained using different initial learning rates (LR) for 700 steps. After a set of experiments where we test the learning rate with different orders of magnitude, the range of values between 0.0001 and 0.0005 leads to an improvement in the mAP. In this range, the value of 0.0002 results in faster convergence.

As shown in Fig 7, changing the optimizer from Momentum to Adam with exponential LR allowed the model to stabilize at around 400 steps, with an approximate mAP value of 0.8, whereas Momentum required 600 steps to achieve that performance. Due to this faster convergence, Adam was selected as the optimizer in the subsequent experiments. Furthermore, with Adam as the optimizer, the initial LR was defined as 0.0002, which seemed to offer better accuracy and to boost the model’s performance in earlier stages, when compared to other LR values, as shown in Fig 8.

#### Changing the anchor generator’s stride

When generating bounding boxes, different parameters should be adjusted to optimize the system’s performance. One of these parameters is the stride (i.e., the space between each anchor). The TensorFlow API defines as default a value of 8 pixels, but this value can be doubled, when needed, to achieve a faster training time.

As seen in Table 6, because were dealing with small cells, a value of 8 pixels was adequate for the problem at hand. When defining this value as 16 pixels, a mAP of 0.8 was achieved, whereas with 8 pixels, the mAP reached a value of 0.85.

**Table 6:** Comparison of detection results with strides of 8 and 16 pixels on the anchor generator.

Stride	mAP at step				Speed/epoch (sec)
	100	200	300	400	
8	0.67	0.82	0.82	0.85	96.77
16	0.76	0.80	0.80	0.82	46.01

Performance comparison using mAP at different stride values applied on the anchor generator (8 and 16 pixels), at 100, 200, 300, and 400 steps, and the training time for one epoch in each scenario. A smaller stride might improve mAP further.

Changing the stride also affects the training time. In particular, decreasing the stride’s value from 16 pixels to 8 pixels seemed to decrease the model’s speed from 46 seconds to 96 seconds.

#### Using an atrous rate

The atrous rate allows object-detection models to detect smaller objects. In this experiment, we tested the model’s performance with and without an atrous rate. As seen in Table 7, using an atrous rate during training provided slightly better results than not using an atrous rate did, and doing so did not affect the training time.

**Table 7:** Comparison of detection results when using an atrous rate.

Atrous rate	mAP at step				Speed/epoch (sec)
	100	200	300	400	
TRUE	0.67	0.82	0.82	0.85	96.77
FALSE	0.63	0.75	0.82	0.84	96.77

Comparison of the mAP values of the models trained in two scenarios: when using an atrous rate (TRUE) and not using one (FALSE). This performance was evaluated at 100, 200, 300, and 400 steps, and the training time per epoch is shown in seconds for each context.

#### Changing the IoU threshold

The IoU threshold defines what bounding boxes should be considered as overlapping during the NMS technique. The chosen threshold value, or cutoff value, defines the models’ level of sensitivity and specificity. The higher the value, the more specific and less sensitive the model, leading to fewer false positives and more true negatives. In contrast, a lower value will translate in higher sensitivity and lower specificity by obtaining more false positives and fewer true negatives. The threshold value, a problem-dependent parameter, was adjusted and optimized using grid search and the one that produced a better evaluation metric on the validation set was chosen.

We evaluated the model’s performance under various IoU thresholds, ranging from 0.3 to 0.8, as seen in Table 8.

**Table 8:** Comparison of detection results using various IoU thresholds, ranging from 0.3 to 0.8.

IoU (threshold)	Total Loss Validation	Box validation loss		mAP	Speed/epoch (sec)
		Loc.	Class.		
0.3	1.15	0.16	0.17	0.84 (317)	102.40
0.4	1.36	0.19	0.40	0.84 (400)	98.09
0.5	1.61	0.33	0.49	0.84 (329)	97.30
0.6	2.17	0.58	0.72	0.83 (332)	96.26
0.7	2.75	0.74	1.02	0.85 (400)	96.77
0.8	2.13	0.57	0.73	0.83 (337)	96.40

Total loss for validation, box-validation loss for localization (Loc.) and classification (Class.), best mAP before 400 steps, and training time for one epoch in seconds using various IoU thresholds (ranging from 0.3 to 0.8). The change in the IoU threshold, although it did not lead to significant changes in mAP, led to the best performance with a value of 0.7.

Decreasing the IoU threshold in NMS increases the number of accepted proposals, which increases training time. However, decreasing the IoU threshold seems to affect the validation losses regarding classification and localization at the second stage.

### Performance evaluation for overcrowded scenes

The maximum number of proposals parameter should always be, at a minimum, the maximum number of ground-truth boxes present in the input images. We tested how the number of proposals influenced the model’s performance by manually adjusting this value from 1000 to 3500 proposals in steps of 500, as seen in Table 9. Increasing the number of proposals increased the training time.

**Table 9:** Validation losses, mAP, and training speed comparison using different numbers of proposals in a range of 1000 to 3500.

Number of proposals	Total loss Val.	Box val. loss		mAP	Speed/epoch (sec)
		Loc.	Class.		
1000	1.67	0.39	0.40	0.83(340)	95.49
1500	1.34	0.25	0.32	0.83(381)	97.56
2000	1.36	0.19	0.40	0.84(400)	98.09
2500	1.29	0.16	0.33	0.83(400)	101.05
3000	1.15	0.11	0.25	0.85(310)	104.34
3500	1.12	0.12	0.20	0.84(213)	106.80

Total loss for validation, box-validation loss for localization (Loc.) and classification (Class.), best mAP until 400 steps, and training time for one epoch in seconds using between 1,000 and 3,500 proposals. Increasing the number of proposals decreased the box validation loss and, consequently, improved the mAP.

Although the final mAP indicates no apparent differences between the number of proposals, the model performed better with 3,000 proposals. Thus, we can conclude that the number of proposals generated can affect the training speed without any significant effects on the model’s accuracy. However, the losses associated with the second stage of the algorithm showed significant reductions when increasing the number of proposals, even though that shrinkage was not visible in the final mAP.

### Dealing with small datasets

In this experiment, we evaluated the effectiveness of various data-augmentation techniques on the model’s performance. We tested the approaches individually for 400 steps (20 epochs). Table 10 shows that the model’s performance increased significantly when applying data-augmentation techniques. In particular, the mAP values increased by 0.3 with respect to the baseline model, which did not use data augmentation.

**Table 10:** mAP value at steps 100, 200, 300, and 400 when using various combinations of data-augmentation techniques.

Data Augmentation	mAP at step			
	100	200	300	400
(00) None	0.12	0.32	0.51	0.59
(01) Horizontal flip	0.51	0.75	0.81	**0.82**
(02) Vertical flip	0.12	0.57	0.78	0.81
(03) Pixel value scale	0.40	0.76	0.78	0.81
(04) Image scale	0.72	0.79	0.80	0.80
(05) RGB to gray	0.47	0.79	0.81	0.81
(06) Adjust brightness	0.68	**0.82**	**0.82**	0.80
(07) Adjust contrast	0.01	0.02	0.29	0.76
(08) Adjust saturation	0.02	0.11	0.66	0.78
(09) Distort color	0.25	0.75	0.80	0.81
(10) Jitter boxes	0.76	0.77	**0.82**	0.79
(11) Crop image	0.67	0.78	0.76	0.80
(12) Crop to aspect ratio	0.36	0.80	**0.82**	0.81
(13) Black patches	0.02	0.40	0.75	0.80
(14) 90º rotation	0.04	0.45	0.76	0.80
(15) Top4: (01, 06, 10, 12)	0.68	0.78	0.78	0.81
(16) All	0.51	0.74	0.81	**0.82**

Performance comparison for the mAP of the models trained using distinct data-augmentation scenarios. This performance was evaluated at 100, 200, 300, and 400 steps, and the training time per epoch is shown in seconds for each context. The set of augmentations implemented should not be random. We tested various methods on the data to understand the techniques to be applied.

The results indicate that the most effective augmentation techniques for short-term training were image scaling, jitter boxes, brightness adjustment, and image cropping. The saturation adjustment proved its efficiency only after more than 300 steps of training. After checking the data-augmentation techniques individually that produced the best performance, we tested two situations: combining the techniques that gave the best results (0.82 mAP) individually, named the Top 4 (15), and testing all augmentation techniques simultaneously (16). Although the mAP of the Top 4 did not differ when compared to the best individual techniques, using a set of data-augmentation techniques can provide results that are better than or comparable to those techniques used in isolation.

### Dealing with objects of different shapes

The grid-anchor generator can be defined by the stride, scales, and aspect ratios. The experiments that followed the evaluation of the augmentation techniques analyzed the behavior of the training according to some changes to those parameters. Notably, at any given location (i.e., at each anchor), the number of the scales times the number of the aspect ratio anchors was generated with all possible combinations of scales and aspect ratios. In this experiment, we optimized the anchor generation process in a quantifiable way to match the receptive field shapes and reduced the number of invalid anchors. Taking as the baseline the cell exploration performed in the Data section, in which the dimensions and the aspect ratio of the different cells were analyzed, we made some changes to the default values used in Faster R-CNN and evaluated how those changes affected performance during the detection stage.

#### Changing the anchor generator’s aspect ratio

By default, Faster R-CNN considers the aspect ratio using three values (0.5, 1.0, and 2.0). In this experiment, we checked whether increasing the number of aspect ratios could also increase the model’s performance. Therefore, we added the value of 1.5, considering that the cells could assume different shapes and could demand more flexible aspect ratios. As the results in Table 11 show, adding one more aspect ratio to the anchor generator did not improve the model’s performance in the long term. Although the mAP rose faster when using this additional value, we verified that using only three aspect ratios was sufficient to detect the objects in our images. This could be explained by the fact that the bounding boxes are eventually readjusted to the ground-truth boxes using the localization losses in the first and second stages of the object detection.

**Table 11:** Comparison of detection results for three and four aspect ratio values.

Aspect ratios	mAP at step				Speed/epoch (sec)
	100	200	300	400	
[0.5, 1.0, 2.0]	0.51	0.74	0.81	0.82	94.99
[0.5, 1.0, 1.5, 2.0]	0.74	0.80	0.80	0.82	96.00

Performance comparison by mAP of the models trained using two possible aspect ratios. This performance was evaluated at 100, 200, 300, and 400 steps, and the training time per epoch for each configuration is shown in seconds. In object detection systems, the bounding boxes in an image can assume different shapes, reflected by the aspect ratios of those bounding boxes. An additional aspect ratio was used to create initial bounding boxes that could potentially improve the fit to the cells’ morphology.

#### Changing the anchor generator’s scale

Image resizing to 1100 × 1100 pixels affects the scale value used in the anchor generator. Therefore, the values presented in Table 2 for the cells’ width, height, distribution should undergo adjustments due to the new dimensions. Table 12 shows those values scaled for the new reality, as well as a descriptive statistical summary.

**Table 12:** Descriptive summary (mean, range and percentiles) of the cells’ dimensions in the scaled images.

Dimension	Mean	Std	Min	25%	50%	75%	Max.
Width	34.2	9.8	9	28	32	41	95
Height	32.6	9.4	6	26	32	39	97

Descriptive summary (mean; standard deviation; minimum; first, second, and third quartiles; and maximum) related to the width and height of the cells present in the input images scaled to 1100 × 1100 pixels. There was a need to adjust the values from Table 2 due to image resizing.

To calculate the best scales to use for the anchors, and because the size basis for the anchors was 256 pixels, the values of the pixels presented on Table 13 were scaled to this size.

**Table 13:** Comparison of detection results using different scales for the anchor generator.

Scale	mAP at step				Speed/epoch (sec)
	100	200	300	400	
[0.25, 0.5, 1.0, 2.0]	0.74	0.80	0.80	0.82	96.00
[0.05, 0.15, 0.3, 0.5]	0.58	0.76	0.79	0.83	97.11
[0.15, 0.3, 0.5]	0.76	0.82	0.81	0.82	95.74
[0.15, 0.3, 0.5, 1.0]	0.67	0.82	0.82	0.85	96.77
[0.3]	0.01	0.02	0.01	0.01	92.82

mAP performance comparison of the models trained using five scales at the anchor generator. This performance was evaluated at 100, 200, 300, and 400 steps, and the training time per epoch is shown in seconds for each configuration. The scales were adjusted so that the cells’ dimensions were taken into account.

The cells’ mean width and height was around 0.13 of the basis anchor size. The minimum was around 0.03, and the maximum was 0.38. Considering these values, we executed experiments with various scales to check the behavior of the mAP at 400 steps.

The results shown in Table 13 indicate that limiting the scales to values similar to the cell size increases overfitting, thus leading to poorer performance. However, reducing the lowest value of the scale improves the model because the value is better adjusted to the problem’s reality.

By testing a single scale (in this case, the value 0.3), we were able to corroborate that the object detection was affected profoundly. After concluding that the scales list [0.15, 0.3, 0.5, 1.0] achieved the best results in the previous experiments, we re-evaluated the mAP using the default list of aspect ratios to determine the best configuration to use in continuing the experiments. By combining the information on scales and aspect ratio, we confirmed, as shown in Table 14, that combination of the aspect ratios [0.5, 1.0, 1.5, 2.0] and scales [0.15, 0.3, 0.5, 1.0] resulted in the best mAP. Following this configuration, each anchor defines 16 bounding boxes.

**Table 14:** Comparison of detection results with various aspect ratios and the scale [0.15, 0.3, 0.5, 1.0].

Aspect ratio	mAP at step				Max mAP (step)
	100	200	300	400	
[0.5, 1.0, 2.0]	0.73	0.76	0.80	0.81	0.82 (238)
[0.5, 1.0, 1.5, 2.0]	0.67	0.82	0.82	0.85	0.85 (400)

Performance comparison by mAP of the models trained using various aspect ratios with the scale [0.15, 0.3, 0.5, 1.0]. This performance was measured at 100, 200, 300, and 400 steps, and we identified the step at which the models achieved the best mAP.

### Evaluating the models’ regression metrics

To select the models in the previous steps, we mainly considered the mAP obtained after training. To evaluate the models further, we evaluated several regression metrics, including the RMSE, MAE, and MEDAE, on the data set with medium complexity. The results are shown in S1–S3 Tables.

After evaluating the results, we selected the six best models and executed each for 2,000 steps to check their performance. The six models were as follows:

1. **Fine-tuned (A)**—Takes into account the model that was fine-tuned to balance mAP and speed over several adjustments. It is represented in blue, and it achieved a mAP of 0.8457.
2. **Best regression (B)**—Takes into account the model that returned the best average regression metrics. It is represented in yellow, and it achieved an mAP of 0.7705.
3. **Mixed regression and fine-tuned (C)**—As a mix between the two previous options, the best regression metric results were chosen only if the mAP did not change by more than 0.01 from the best mAP obtained with the present configuration. It is represented in green, and it achieved a mAP of 0.8300.
4. **Best mAP (D)**—The model that obtained the highest mAP during the experiments. It is represented in magenta and achieved a mAP of 0.8558.
5. **Best RMSE&MAE (E)**—The model that obtained the highest RMSE and MAE during the experiments. It is represented in orange and had a mAP of 0.8293.
6. **Best MEDAE (F)**—The model that obtained the highest MEDAE during the experiments. It is represented in gray, and it had a mAP of 0.8426.

As Table 15 shows, the model with the highest mAP is not necessarily the one with the best regression results.

**Table 15:** Performance comparison of the six best models for the medium level of complexity and for the entire data set.

Model	Medium Level			All levels		
	RMSE	MAE	MEDAE	RMSE	MAE	MEDAE
A	9.8	9.0	9.5	64.5	30.8	10.0
B	122.0	112.1	99.5	223.4	160.4	100.0
C	18.7	15.4	13.5	81.0	41.2	15.0
D	11.2	9.4	10.0	45.1	24.8	11.0
E	**8.8**	**6.4**	**3.5**	52.7	23.7	7.0
F	10.0	7.5	6.0	**24.8**	**17.4**	**13.0**

Performance comparison of the six final models using regression metrics, specifically the root mean squared error (RMSE), mean absolute error (MAE), and median absolute error (MEDAE). This evaluation was conducted using the entire data set (all levels) and those images considered to have medium complexity (medium level).

Upon noticing the different effects of image complexity on the final architectures, we defined three models for each level and adjusted the score threshold.

For the low-complexity images, we chose the best MEDAE model and defined the score threshold as 0.9. For the medium level, we selected the best RMSE & MAE model, and a threshold of 0.4. Finally, for the high-complexity images, we selected the best RMSE & MAE model, and a threshold of 0.2.

Finally, descriptive statistics were inferred from the final results, as shown in Table 16, and conclusions were made.

**Table 16:** The range, mean, and percentiles of error associated with the various complexity levels.

Level	[Min,Max]	Mean ± STD	25%	50%	75%
Low	[0,10]	5.86±3.58	3	6	10
Medium	[1,45]	14.37±13.12	3.25	6	22.5
High	[1,79]	35.00±32.13	7	22	68
All	[0,79]	16.53±20.96	3.75	6.5	22.25

Descriptive summary statistics (minimum; maximum; mean; standard deviation; and first, second, and third quartiles) describing the error encountered in the subsets of images defined by complexity level.

The results were as follows:

For the low level, the error found in samples was in the range of [0, 10] units, with a standard deviation of 3.58. Half of the samples had fewer than 6 units of error.
For the medium level, the error range was [0, 45], with a standard deviation of 13.12. Half of the samples had fewer than 6 units of error, and 75% had fewer than 22.5 units of error.
3. For the high level, the error range was [1, 79], with a standard deviation of 32.13. Half of the samples had fewer than 22 units of error.
4. Overall, the samples’ error range was [0, 79], with a standard deviation of 20.96. Half of the predicted values had an absolute difference from the real values of lower than 6.5 units of error.

S3–S5 Figs present the inference of the three levels as examples, alongside the originally labeled ones.

## Conclusion

This paper proposes a refined version of Faster R-CNN (S6 Fig) for automatic counting of cancer cells with a promising mAP. We overcame image constraints such as small objects, overcrowded scenes, and small data sets by carefully adjusting distinct hyperparameters included in this process. We showed that object detection is a potentially effective and efficient counting technique that could lead to good results, even given weak labeling of the ground truth. In future work, we will attempt to expand this process to other cell types that have similar problems. Applying the insights obtained during this project to a new, larger data set will eventually lead to models with higher accuracy. Moreover, at least three labeled data sets with significant numbers of images should be defined, taking into account the images’ complexity. As perceived during the experiments, the model’s performance in inferring counts is positively associated with the clump of cells present in the images. This should be considered in future works.

## Supporting information

S1 Fig Architecture of Inception ResNet V2. Architecture of Inception ResNet V2, developed during the course of this research.
(TIF)

S2 Fig Colorectal zebrafish xenografts. Colorectal cancer cells were labeled using a lipophilic die (DiI-red) and injected into two-days -post-fertilization zebrafish embryos and fixed for immunofluorescence and confocal imaging at four days post injection. Immunofluorescence was performed to detect apoptosis (Activated Caspase3- green) and proliferation (Ki-67-white), and nuclei were counterstained with DAPPI (blue). A-B. Montage of a confocal z stack of CRC zebrafish xenograft. C. Manual counting of one slice using the manual Cell Counter plugin in Fiji.
(TIF)

S3 Fig Ground-truth labeling and inferred detections for low-complexity images. Comparison between the ground truth and the inferred detections in low-complexity images. On the left is an image labeled by a medical expert. The results of the trained model are provided on the right.
(TIF)

S4 Fig Ground-truth labeling and inferred detections for medium-complexity images. Comparison between the ground truth and the inferred detections in medium-complexity images. On the left is an image labeled by a medical expert. The results of the trained model are provided on the right.
(TIF)

S5 Fig Ground-truth labeling and inferred detections for images with high complexity. Comparison between the ground truth and the inferred detections in high-complexity images. On the left is an image labeled by a medical expert. The results of the trained model are provided on the right.
(TIF)

S6 Fig Overview of the implemented object-detection system. Overview of the Faster R-CNN object-detection system implemented in this research. This system includes a first stage (RPN) whose main purpose is to propose a set of regions where objects could be present, and a second phase in which the output of the first phase is used to detect and classify objects in those regions.
(TIF)

S1 Table Regression metrics and mAP values for the various models: Training and data augmentation. Table with the resulting regression metrics and mAP values for various configurations of training and-data augmentation parameters. The asterisk (*) represents the default model for Faster R-CNN.
(TIF)

S2 Table Regression metrics and mAP values for the various models: First stage. Table with the resulting regression metrics and mAP for various configurations of first-stage parameters. The asterisk (*) represents the default model for Faster R-CNN.
(TIF)

S3 Table Regression metrics and mAP values for the various models: Second stage. Table with the resulting regression metrics and mAP for various configurations of second-stage parameters. The asterisk (*) represents the default model for Faster R-CNN.
(TIF)
