# Supplementary figures and images for: Object detection for automatic cancer cell counting in zebrafish xenografts
Source: PLoS One. 2021 Nov 29;16(11):e0260609. doi: 10.1371/journal.pone.0260609 (PMC8629215; doi:10.1371/journal.pone.0260609)

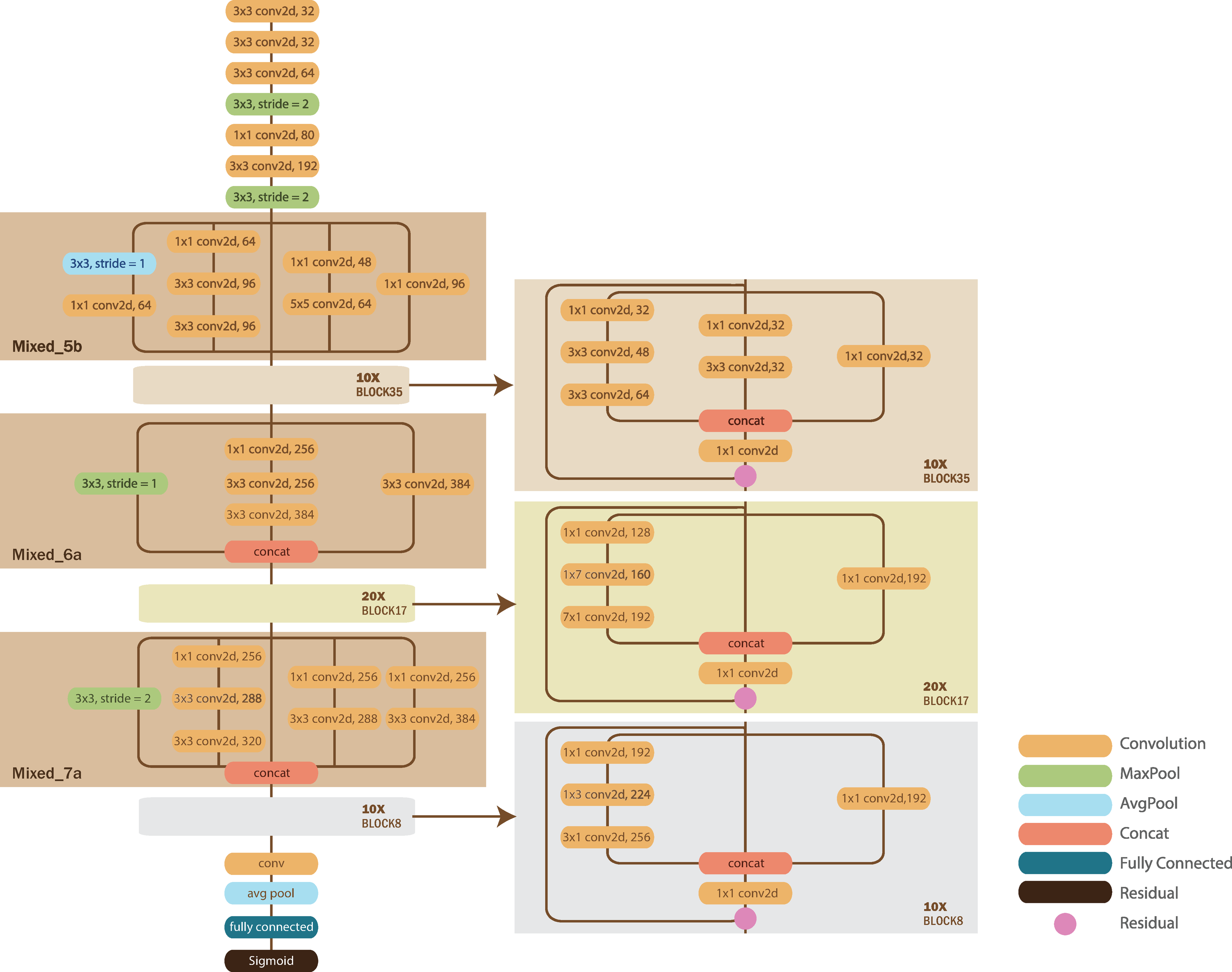

Supplement: S1 Fig — Architecture of Inception ResNet V2, developed during the course of this research. (TIF) [file pone.0260609.s001.tif]

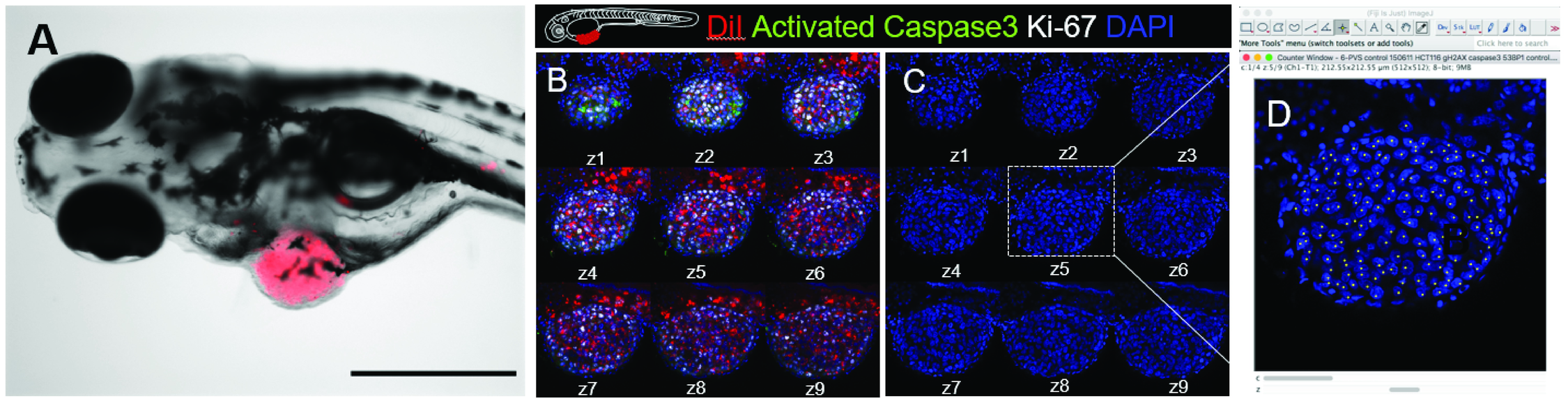

Supplement: S2 Fig — Colorectal cancer cells were labeled using a lipophilic die (DiI-red) and injected into two-days -post-fertilization zebrafish embryos and fixed for immunofluorescence and confocal imaging at four days post injection. Immunofluorescence was performed to detect apoptosis (Activated Caspase3- green) and proliferation (Ki-67-white), and nuclei were counterstained with DAPPI (blue). A-B. Montage of a confocal z stack of CRC zebrafish xenograft. C. Manual counting of one slice using the manual Cell Counter plugin in Fiji. (TIF) [file pone.0260609.s002.tif]

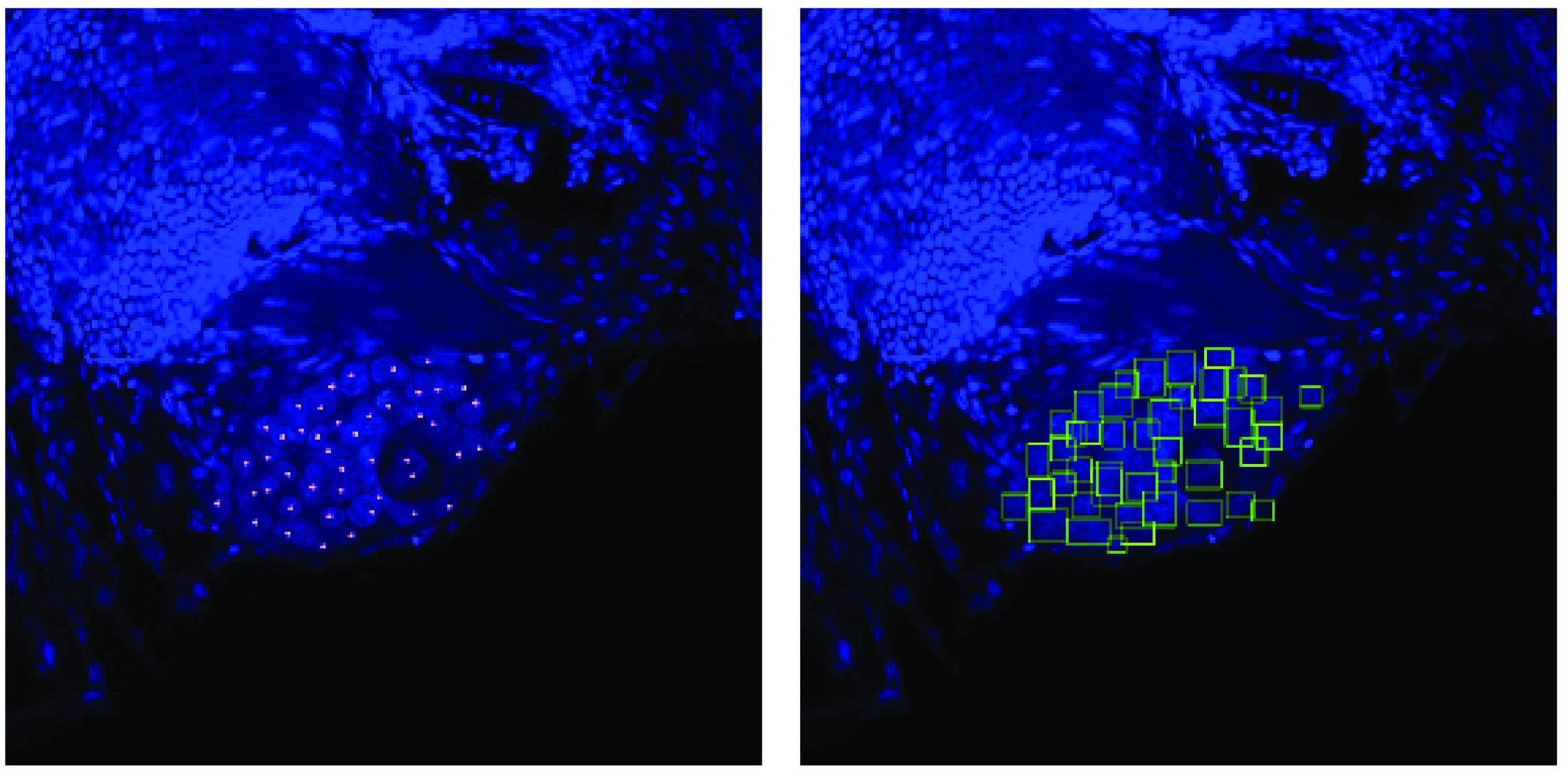

Supplement: S3 Fig — Comparison between the ground truth and the inferred detections in low-complexity images. On the left is an image labeled by a medical expert. The results of the trained model are provided on the right. (TIF) [file pone.0260609.s003.tif]

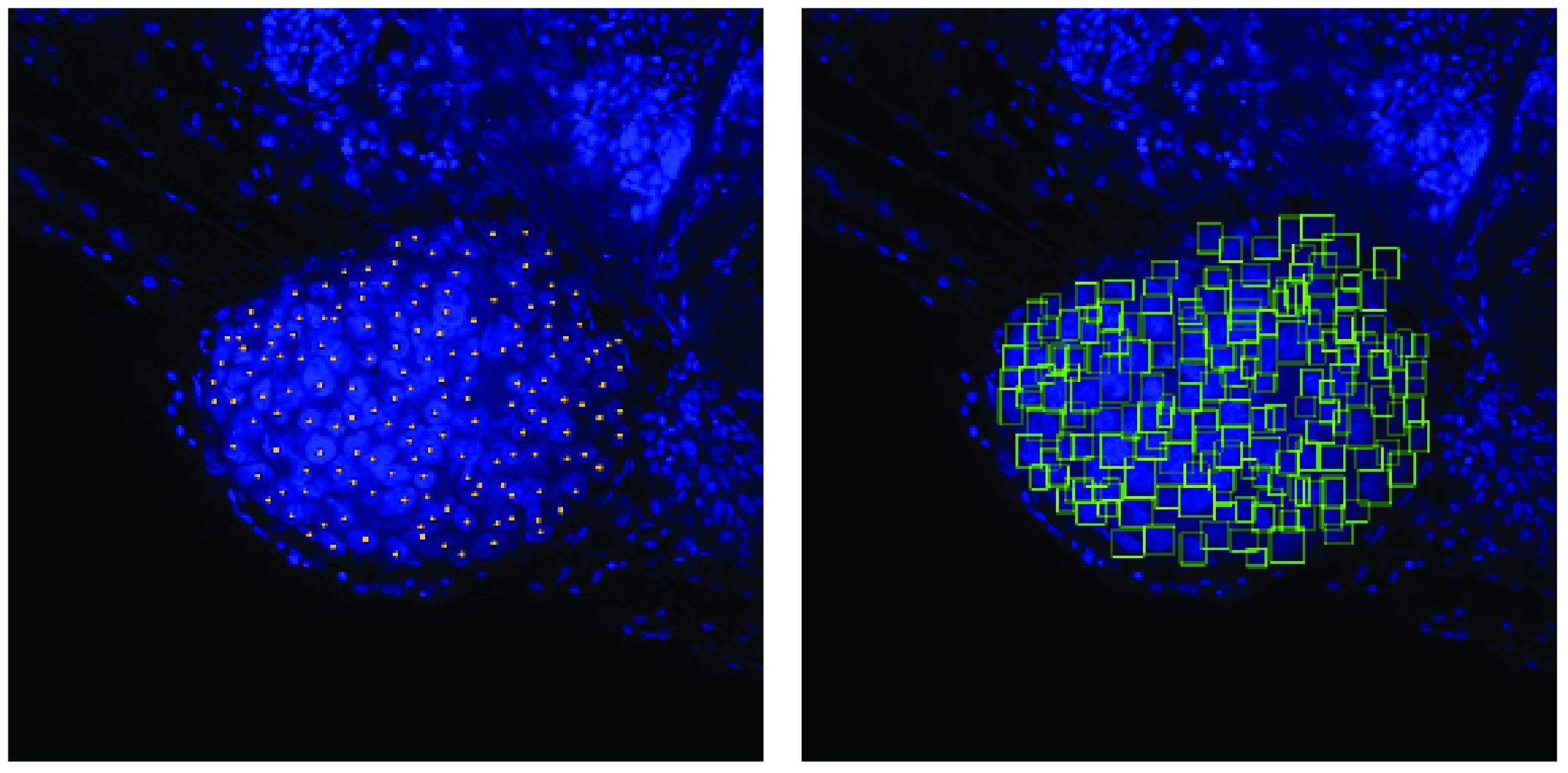

Supplement: S4 Fig — Comparison between the ground truth and the inferred detections in medium-complexity images. On the left is an image labeled by a medical expert. The results of the trained model are provided on the right. (TIF) [file pone.0260609.s004.tif]

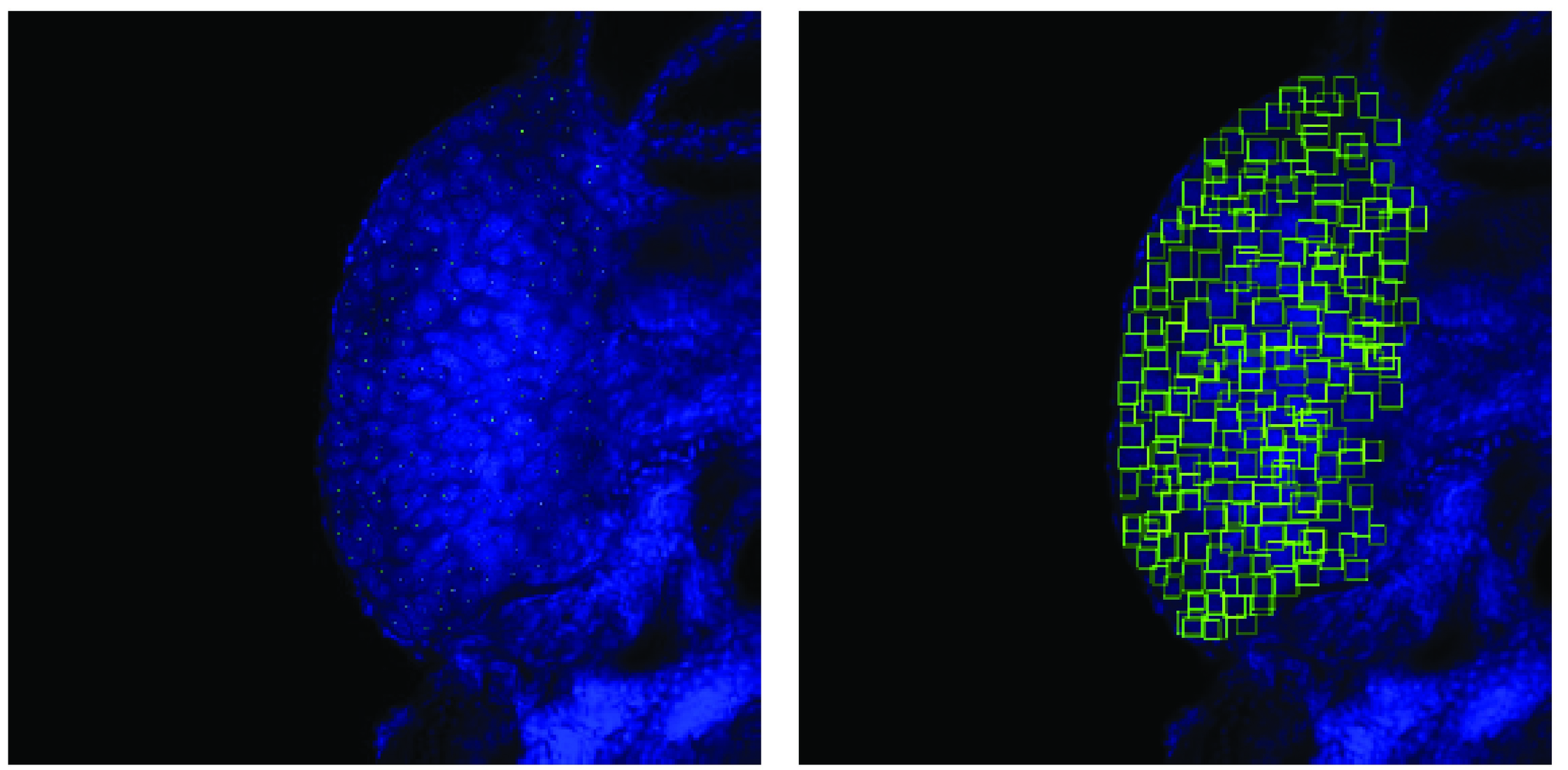

Supplement: S5 Fig — Comparison between the ground truth and the inferred detections in high-complexity images. On the left is an image labeled by a medical expert. The results of the trained model are provided on the right. (TIF) [file pone.0260609.s005.tif]

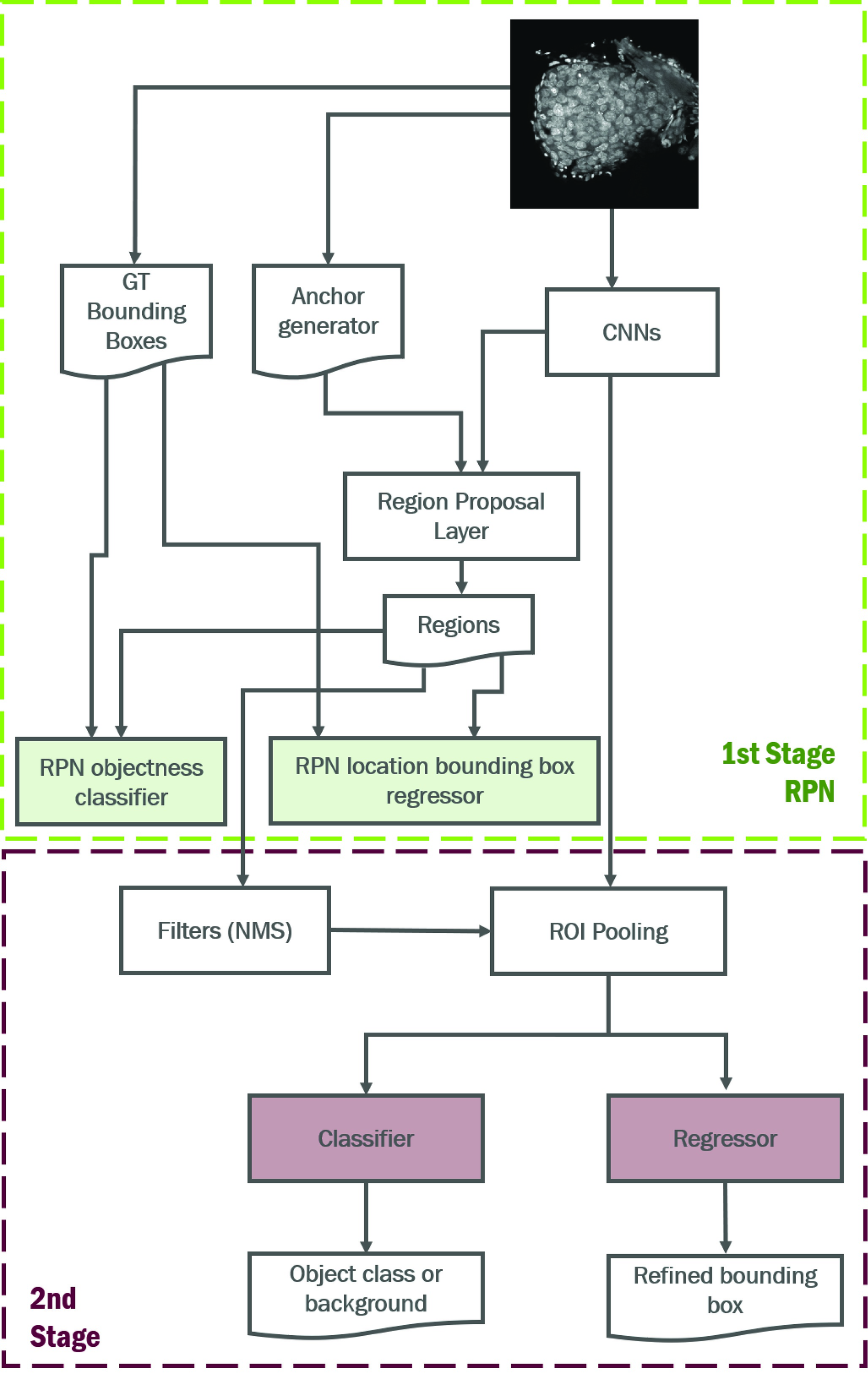

Supplement: S6 Fig — Overview of the Faster R-CNN object-detection system implemented in this research. This system includes a first stage (RPN) whose main purpose is to propose a set of regions where objects could be present, and a second phase in which the output of the first phase is used to detect and classify objects in those regions. (TIF) [file pone.0260609.s006.tif]

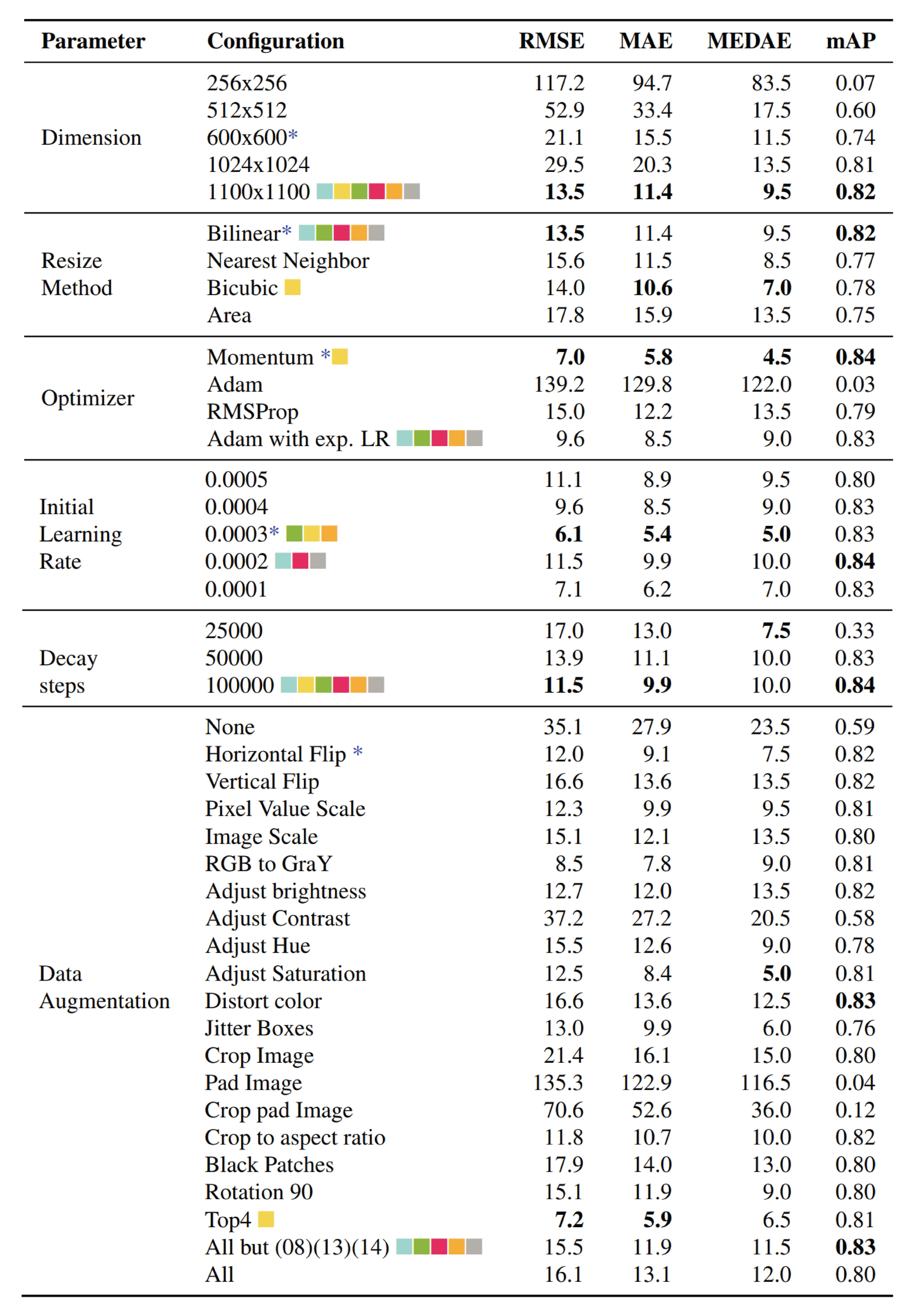

Supplement: S1 Table — Table with the resulting regression metrics and mAP values for various configurations of training and-data augmentation parameters. The asterisk (*) represents the default model for Faster R-CNN. (TIF) [file pone.0260609.s007.tif]

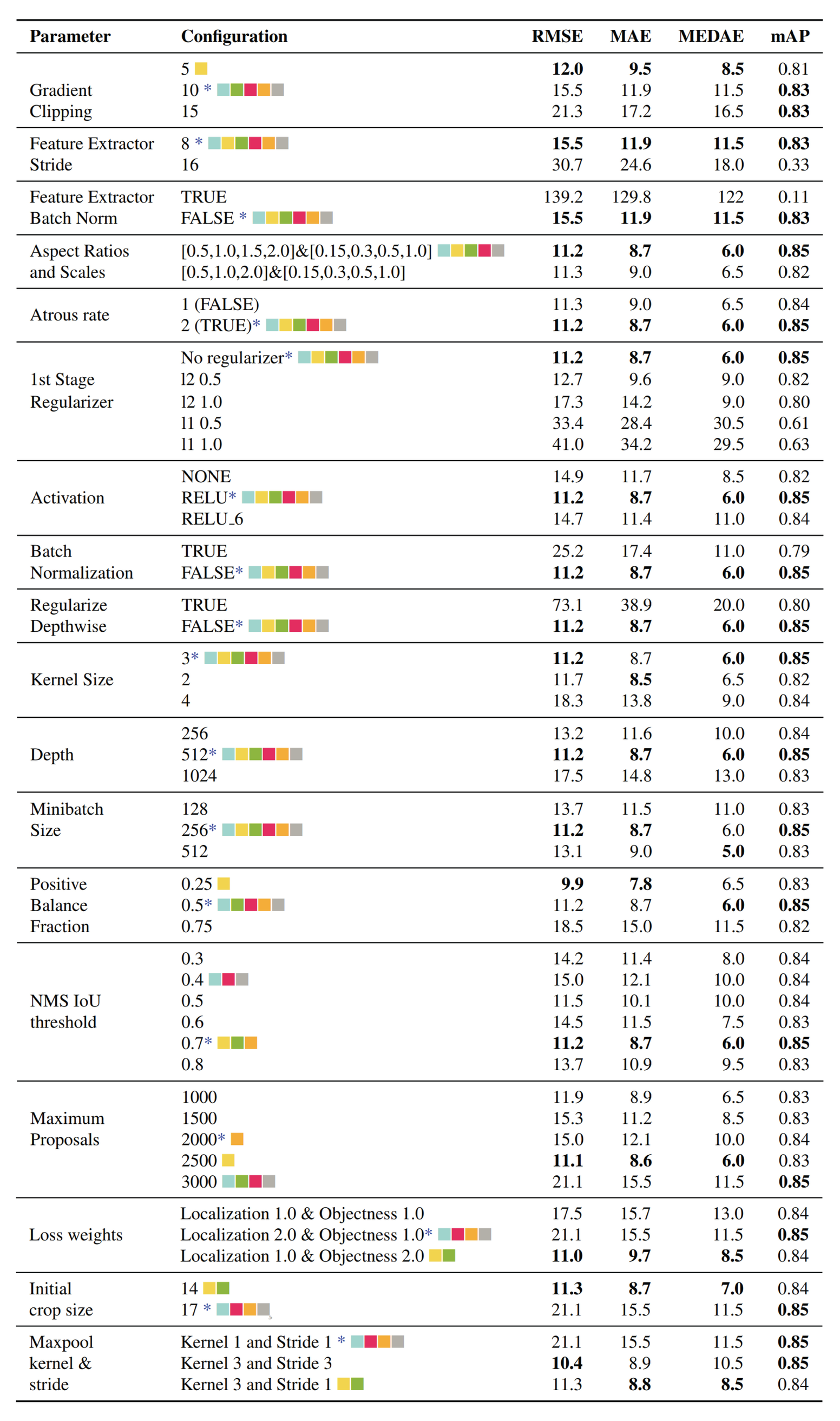

Supplement: S2 Table — Table with the resulting regression metrics and mAP for various configurations of first-stage parameters. The asterisk (*) represents the default model for Faster R-CNN. (TIF) [file pone.0260609.s008.tif]

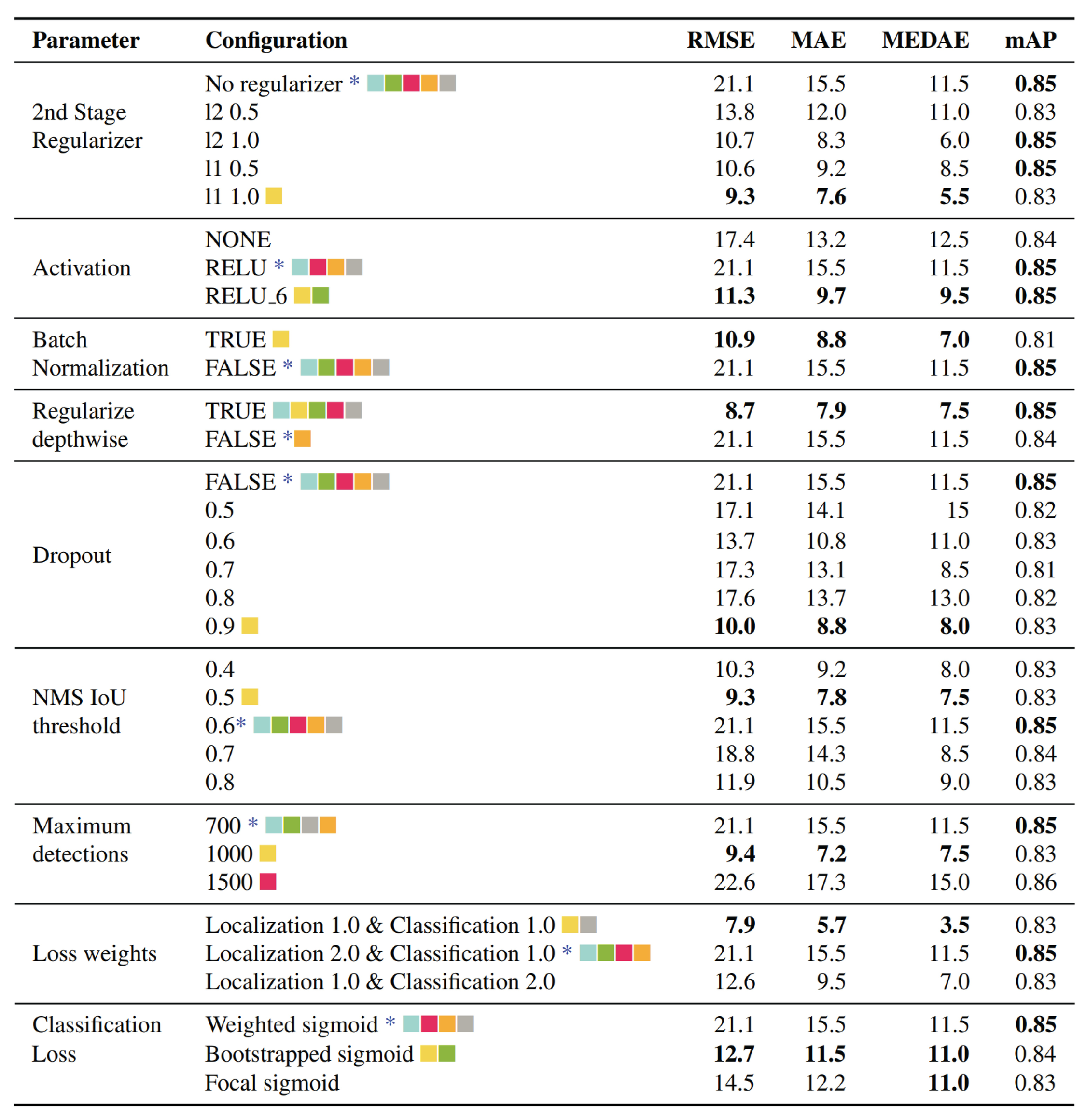

Supplement: S3 Table — Table with the resulting regression metrics and mAP for various configurations of second-stage parameters. The asterisk (*) represents the default model for Faster R-CNN. (TIF) [file pone.0260609.s009.tif]
